# Supplementary material for: Global Neuropsychopharmacological Prescription Trends in Adults with Schizophrenia, Clinical Correlates and Implications for Practice: A Scoping Review
Source: Brain Sci. 2023 Dec 20;14(1):6. doi: 10.3390/brainsci14010006 (PMC10813099; doi:10.3390/brainsci14010006)
Supplement: Supplementary file 1 [file brainsci-14-00006-s001.zip › brainsci-2780783-supplementary.pdf]

Table S1. Summary of details of all included studies in this review

No.	Author / year / country (region)	Nature and goal of study	Participants and demographic features	AP prescribed	MS prescribed	AD prescribed	Main Correlates / Main Findings
1	Tamene et al / 2023 / Ethiopia [1]	Cross-sectional study<br><br>To assess APP and its associated factors among SZ patients	N = 422<br>Male 41.7%<br>Age 36.4 ± 11.5	APP 22.7%<br><br>Among those on APP, 80.2% used combined FGA<br><br>24.4% took haloperidol<br><br>13.5% took a combination of haloperidol and fluphenazine decanoate		34.5% took amitriptyline	APP a/w:<br>Longer duration of illness<br>Longer duration of treatment<br>Greater number of admissions<br>Substance use
2	Miron et al / 2023 / Romania [2]	Cross-sectional study<br><br>To investigate the concomitant use of BZD and MS among stabilized SZ patients	LAI group:<br>N = 77<br>Male 44.15%<br>Age 52.92 ± 12.24<br><br>Oral AP group:<br>N = 238<br>Male 40.34%<br>Age 51.32 ± 11.10	24.44% on LAI<br><br>75.56% on oral AP<br><br>43.17% on AP monotherapy	26.66% on MS		37.77% on BZD<br><br>Patients on SGA LAI more likely to be on monotherapy

3	Luthra et al / 2023 / Australia [3]	Cross-sectional study<br><br>To determine the prevalence of high-dose antipsychotic prescribing in adult patients with schizophrenia	N = 358<br>Male: 76%<br>Age: adult patients	15% high dose AP<br><br>20% APP<br><br>CLZ: 25%			High dose AP a/w:<br>Male<br>Indigenous people<br>Being under Mental Health Act/ involuntary treatment
4	Fulone et al / 2023 / Brazil [4]	Retrospective cohort study<br><br>To investigate sociodemographic and clinical characteristics of users of atypical AP for the treatment of SZ	N = 522071<br>Male 50.9%<br>Age: 19 to 59	CLZ 5.8%<br>Olanzapine 34.1%<br>Risperidone 30.3%<br>Quetiapine 11.8%<br>Ziprasidone 3.2%			Olanzapine was the most prescribed AP in adults
5	Fond et al / 2023 / France [5]	Population-based study<br><br>To describe the long-term management of patients with SZ treated with AP in real-world practice	N = 456003<br>Male 53.7%<br>Age ≥ 18 years	96% given oral AP,<br>17.5% given FGA LAI<br>16.1% given SGA LAI<br><br>Persistence rate at 24 months for oral AP: 23.9%, for FGA LAI: 11.5%, for SGA LAI: 20.8%	MS use 28.5%	AD use 45.7%	Anxiolytics use 62.1%<br><br>MS, AD, anxiolytics use a/w:<br>Women<br>Aged ≥ 50 years

6	Lagreula et al / 2022 / Belgium [6]	Retrospective observational study<br><br>To explore APP and other psychotropic prescribing patterns before and after psychiatric hospitalisations for patients with SZ & SZA	N = 516<br>Male 55.4%<br>Age 40 ± 11.6 years<br>(Discharged from hospital <1 year, on at least 1 AP at admission)	APP increased significantly from 47.9% on hospital admission to 59.1% at discharge<br><br>CLZ use increased from 9.3% to 11%	Adjunctive MS use increased from admission to discharge (16.9% vs 19.6%)	Adjunctive AD use: no significant change from admission to discharge (30.6% vs. 33.1%)	APP on admission a/w:<br>Previous trial of ≥2 different AP<br>Prior CLZ use<br>Treatment with an FGA<br>↑ PDD/DDD ratio on admission<br>↑ Number of hypno-sedatives<br>↓ Involuntary admission<br><br>APP at discharge a/w:<br>Prior CLZ use<br>Treatment with a FGA<br>↑ PDD/DDD ratio on admission<br>↑ Number of hypno-sedatives<br>↓ Involuntary admission<br>↓ Treatment with trazodone/a sedating antihistamine<br><br>Adjunctive BZD use increased from admission to discharge (45.5% to 54.3%)<br><br>Adjunctive anticholinergic use increased from admission to discharge (9.3% vs. 11.6%)
7	Vadiei et al / 2022 / United States [7]	Retrospective, cross-sectional study<br><br>To determine patterns and predictors of oral AP prescribing for adults diagnosed with SZ/SZPH	N = 38403 (unweighted n = 1932; age ≥ 18)	70.1% were prescribed AP<br><br>61.7% given SGA<br><br>8.9% given FGA<br><br>16.3% risperidone<br>13.7% olanzapine<br>13.6% quetiapine<br>6.2% CLZ			SGA prescription a/w (vs. no APP):<br>Age <65<br>↓ Number of chronic conditions<br>↑ Number of medications<br>Being seen for a routine visit<br><br>FGA prescription a/w (vs. no APP):<br>↓ Number of chronic conditions<br>Being seen for a routine visit

8	Hori et al / 2022 / Japan [8]	Cross-sectional study<br><br>Investigate the characteristics of the use of anticholinergics in patients with SZ only	N = 2027<br>Male 45.6%<br>Age 45.7 ± 15.2 years	Average daily dose of AP: 683.5 ± 449.1 mg/d CPZeq<br><br>44.1% APP	35.6% on adjunctive MS	8.5% on adjunctive AD	30.5% on adjunctive anticholinergics<br><br>Higher anticholinergic prescription rate a/w:<br>High AP doses (≥1000mg/day CPZeq)<br>APP<br>FGA monotherapy (vs. SGA monotherapy)<br><br>Anticholinergic dosage higher in:<br>High AP doses<br>APP<br>FGA use
9	Anozie et al / 2022 / Nigeria [9]	Cross-sectional study<br><br>To determine the prevalence of high-dose AP and its association in patients with SZ&SZA	N = 320<br>Male 47.8%<br>Age 37.1 ± 10.1 year	38.4% high-dose AP (PDD/DDD > 1.5)<br><br>8.1% high-dose AP (BNF > 100%)<br><br>2.2% high-dose AP (CPZeq >1000mg)			High-dose AP prescription a/w:<br>APP<br>Anticholinergic use<br>↑ Duration of treatment<br>Having current psychotic episode<br>↑ Cost of medication<br>↑ Hormonal side effects

10	Pai et al / 2022 / Australia [10]	Retrospective cohort study<br><br>To examine real-world patterns of AP use in patients with SZ in Australia who initiated treatment for the first time 2013 to 2017	N = 6740<br><br>Male 62%<br><br>Age range 16 – 85 years	First-line:<br>Oral AP 71%<br>FGA LAI 17%<br>FGA oral 7%<br>SGA LAI 3%<br>CLZ 2%<br><br>Second line or later:<br>Oral AP 21%<br>FGA LAI 34%<br>FGA oral 16%<br>SGA LAI 21%<br>CLZ 34%<br><br>4% - 7% increase in proportion of SGA LAI prescriptions from 2013 to 2017<br><br>13% - 19% increase in proportion of FGA LAI prescriptions from 2013 to 2017<br><br>End of study:<br>25.1% on 2 AP<br>2.8% on 3 AP			The median time from the initial prescription to the cessation of that treatment:<br>7.9 months for oral SGA<br>12.7 months for SGA LAI<br>8.8 months for FGA LAI<br>6.2 months for oral FGA<br><br>Persistence to SGA LAI and CLZ is better than for other treatment modalities<br><br>Medication switching was lowest in those on CLZ, and highest in those on oral FGA<br><br>Higher rates of FGA (LAI/oral) use in those ≥45 years old<br><br>Higher rates of CLZ use in those ≥45 years old<br><br>Higher rates of SGA LAI use:<br>Younger age

11	Guo et al / 2021 / China [11]	Cross-sectional study<br><br>Aim to examine prescription pattern for outpatients with SZ in a tertiary hospital	N = 1940<br>Male 46.3%<br>Age 43.3 ± 16.4 years	75.8% on AP<br>24.2% not on AP<br><br>63.3% on SGA<br>10.4% on FGA 2.1% on both FGA and SGA<br><br>63.8% on AP monotherapy<br>12.0% on ≥2 AP<br><br>5.1% on LAIs (out of which 43.4% on SGA LAI)	6.2% on adjunctive MS	15.8% on adjunctive AD	In patients <18 years, highest rate of AP prescription was amisulpride (9.29%)<br><br>In patients 18-65 years, highest rate of AP prescription was risperidone (82.7%)<br><br>26.1% on adjunctive BZD<br><br>APP a/w:<br>↑ Duration of illness<br>↑ LAI use<br>↑ Self-paying cost
12	Gamon et al / 2021 / Spain [12]	Retrospective cohort study<br><br>Aim to assess the appropriateness of AP medications administered to SZ patients and describe current treatment patterns for SZ & SZA	N = 19718<br>64.7% male<br><br>41.3% were new users of AP<br>58.7% were prevalent users of AP	30.4% on ≥2 AP<br><br>9.1% on ≥3 AP<br><br>1.6% exceed maximum allowed dose<br><br>12.5% on FGA monotherapy<br><br>53.9% on SGA monotherapy<br><br>2.1% on FGA polytherapy<br><br>14.7% on SGA polytherapy<br>1313.6% on FGA + SGA<br><br>3% on CLZ<br><br>1.6% on CLZ monotherapy<br><br>1.3% on AP + CLZ	19.1% on adjunctive anticonvulsants<br><br>3.4% on adjunctive lithium	24.5% on adjunctive AD	21.5% used concomitant psychotropics without an associated psychiatric diagnosis.<br><br>APP in prevalent users a/w:<br>Being male<br>Absence of anxiety or depression<br>Absence of alcohol abuse<br>Absence of delirium<br>On antiparkinson, anxiolytic, hypnotic drugs, or lithium<br>↑ Visits to mental health hospital and mental health outpatient clinics<br><br>APP in new users a/w:<br>Being male<br>Absence of anxiety or depression<br>Drug abuse<br>↑ Psychiatric comorbidity (other than anxiety and depression)<br>On antiparkinson, anxiolytic, or hypnotic drugs

							↑ Emergency visits<br>↓ Hospitalizations (for any reason)<br>↑ Visits to mental health hospital<br>↓ Visits to outpatient mental health clinic<br>↓ Visits to non-mental health outpatient clinic
13	Taipale et al / 2021 / Sweden & Finland [13]	Nationwide register-based cohort study<br><br>To compare prevalence of AP and adjunctive pharmacotherapy use among individuals with SZ & SZA between 2006–2016 in Sweden & Finland	Sweden:<br>N = 25433<br>Male 56.7%<br>Age 52.1 ± 12.1 years<br><br>Finland:<br>N = 37780<br>Male 57%<br>Age 56.3 ± 14.7 years	FGA use decreased in both countries, SGA use increased towards 2016<br><br>Oral olanzapine was the most frequently prescribed in 2016 (22.7% in Finland, 20.9% in Sweden)<br><br>CLZ was 2nd most common drug in both countries in 2016 (22% in Finland, 14.8% in Sweden)<br><br>Quetiapine was 3rd most common drug in Finland (17.8%)<br><br>Aripiprazole was 3rd most common drug in Sweden (11.6%)<br><br>78.9% AP use in Finland in 2016<br>77.5% AP use in Sweden in 2016	Adjunctive MS:<br>Sweden:<br>MS 17.3%<br>Lithium 7.8%<br><br>Finland:<br>MS 18.4%<br>Lithium 4.4%	Adjunctive AD:<br>Sweden<br>29.7%<br><br>Finland<br>25.1%	Use of adjunctive BZD and related drugs:<br>32.8% Sweden<br>22.3% Finland<br><br>Increased BZD use in Finland (from 22.3% to 28.4%)<br><br>AD, BZD and Z-drug use were more common among those on APP (vs. those on AP monotherapy)

				Sweden:<br>Oral 65.8%<br>LAI 21.6%<br>FGA LAI 14.3%<br>SGA LAI 7.5%<br>APP 29.2%<br><br>Finland:<br>Oral 72.9%<br>LAI 12.8%<br>FGA LAI 4.3%<br>SGA LAI 8.6%<br>APP 32.6%			
14	Puranen et al / 2020 / Finland [14]	Register-based cohort study<br><br>To investigate adjunctive AD and MS initiation and factors associated in first-episode SZ & SZA	N = 7667<br>Male 56.8%<br>Age 40.2 ± 18.2 years	-	14.1% initiated adjunctive MS within 3 years from diagnosis of SZ<br><br>Valproic acid was the most often initiated MS (8.5%)	35.4% initiated adjunctive AD within 3 years from diagnosis of SZ	Higher risk of adjunctive AD initiation a/w:<br>Female<br>↓ Age<br>BZD and z-drug use<br>On MS<br>On anticholinergic antiparkinson drugs<br>Previous suicidality<br>No previous psychoses episodes requiring inpatient care<br><br>Higher risk of adjunctive MS initiation a/w:<br>Female<br>↓ Age<br>AD use<br>BZD and z-drug use<br>Substance abuse<br>Previous suicidality

							Had one or several psychoses that required hospitalizations
15	Hata et al / 2020 / Japan [15]	Register-based study<br><br>To describe the use of AP in SZ patients to clarify the gap between clinical guidelines and health care practice in Japan trending from 2005 to 2016	N = 12382<br>Male 40.1%<br>Age 40 (median)	80% of patients CPZeq <600mg<br><br>LAIs < 5%<br>CLZ 0.2%<br><br>Aripiprazole increased in popularity, most commonly prescribed in 2016 (31.9%)<br><br>Risperidone and haloperidol use declined<br><br>SGA monotherapy increased from 58.4% in 2005 to 70.8% in 2016<br><br>SGA polytherapy increased from 11.8% in 2005 to 22.1% in 2016<br><br>FGA monotherapy and polytherapy decreased over time<br><br>Approximately 80% of patients on monotherapy (SGA/FGA) during study period	-	-	Use of AP in Japan mostly corresponds to various guidelines, but limited use of CLZ and LAI identified

16	Takahashi et al / 2020 / Japan [16]	Cross-sectional study<br><br>Aim to identify high-dose AP prescription and its associations in patients with SZ	N = 13471<br>Male 44.9%	Mean AP dose 368.3mg/day CPZeq<br><br>21.9% on ≥600mg/day CPZeq (maintenance dose for SZ)<br><br>8.5% on high-dose AP (≥1000mg/day CPZeq)<br><br>26.7% received less than effective dose (<100mg CPZeq per day)	22.1% on adjunctive MS	19.6% on adjunctive AD	58% on adjunctive anxiolytics/hypnotics<br><br>High-dose AP prescription (≥1000mg/day CPZeq) a/w:<br>Males aged 45-49<br>No cerebrovascular diseases<br>On adjunctive anxiolytics/hypnotics<br>Not on adjunctive AD<br>On adjunctive MS<br>On adjunctive antiparkinson drugs<br>Not receiving psychiatrist's therapy<br><br>High-dose AP prescription (≥1000mg/day CPZeq) in older individuals only a/w:<br>On adjunctive anxiolytics/hypnotics<br>Not on AD<br>On adjunctive antiparkinson drugs
17	Ichihashi et al / 2020 / Japan [17]	Cross-sectional study<br><br>Baseline prescription status of treating patients with SZ	N = 1164<br>Male 43.2%<br>Age 46 ± 15 years	43% APP<br><br>57.1% AP monotherapy<br><br>15.5% on AP monotherapy without other psychotropics<br><br>8.3% on LAI<br><br>7.1% on CLZ	37% on adjunctive MS	8% on adjunctive AD	68% on adjunctive anxiolytics/hypnotics

18	Lim et al / 2020 / Asian countries [18]	Cross-sectional study<br><br>To examine co-prescription and dosage of MS with AP in SZ within an Asian research consortium (REAP)	N = 3557<br>Male 59%<br>Age 39.9 ± 12.8 years	SGA twice as prevalent as FGA (80% vs. 39.8%)<br><br>APP 40.1% (more prevalent with adjunctive MS)<br><br>428 ± 358mg/day CPZeq (higher dose with adjunctive MS)	Adjunctive MS: 13.6% (12.8% on one MS, 0.76% on 2 MS, 0.03% on 3 MS)<br><br>11.1% sodium valproate<br><br>1.9% lithium<br><br>Average dose of MS: 613 ± 456 mg/day Li-eq	-	Adjunctive MS a/w:<br>Female<br>↓ Age<br>Country<br>Current hospitalization<br>↑ Duration of illness<br>Disorganized speech<br>Verbal aggression<br>Lack of hallucinations<br>Social-occupational dysfunction<br>Affective symptoms<br><br>Characteristics of those on high-dose MS (>1000mg/day Li-eq) (vs. those on lower MS doses):<br>Treated with FGA<br>Social-occupational dysfunction<br>Disorganized speech<br>↑ AP dose<br>Non-remission of illness
19	Shenoy et al / 2020 / India [19]	To assess the frequency of polypharmacy, reasons for initiation and the factors associated with it in patients with SZ	N = 529	43.9% on APP<br><br>56.1% on AP monotherapy<br><br>23.4% FGA + SGA<br>18.5% SGA + SGA<br>1.1% FGA + FGA<br><br>36.1% on 2 APs<br>6% on 3 APs<br><br>APP almost doubled from 34.2% in 2011 to 61.2% in 2017, followed by a decline to 46.3% in 2018			APP a/w:<br>Extrapyramidal symptoms<br>Excessive sedation<br>↑ AP dose<br>Use of LAI<br>ECT<br>Poor treatment compliance<br><br>Top reasons for APP:<br>37.1% Using a different mode of administration (oral + LAI)<br>17.7% Enhance or augment response of first AP when it was not adequate to manage symptom<br>9.5% Treatment of different symptom domains

							Characteristics of those on APP (vs. AP monotherapy):<br>↑ EPS<br>↑ Excessive sedation<br>↑ Discontinuation of medications due to severe side effects<br>↑ Weight gain<br>↑ Received ECT<br>↑ on LAI<br>↑ Mean AP dose<br>↑ Poor treatment compliance
20	Anozie et al / 2020 / Nigeria [20]	Examined the pattern of AP prescription and polypharmacy among outpatient attendees with SZ in a Nigerian psychiatric hospital	N = 320<br>Male 47.5%	Most commonly prescribed AP (% of total number of prescriptions): 33.1% olanzapine<br>24.4% risperidone<br>21.6% trifluoperazine<br><br>Out of total number of participants:<br>50.9% APP<br><br>4.1% on 3 AP<br>26.6% on FGA + SGA<br>24.4% on FGA + FGA	0.9% on adjunctive MS	4.4% on adjunctive AD	Characteristics of those on APP (vs. AP monotherapy)<br>Males<br>Unmarried<br>Longer duration of illness<br>Adjunctive anticholinergic agents<br>Twice-daily AP dosing interval<br>Alcohol use<br>AD use<br>Current episode of SZ<br><br>Top reasons for APP:<br>66.7% for augmentation/maintenance<br>12.9% due to worsening severity of illness

21	Dong et al / 2019 / Asian countries [21]	Cross-sectional study<br><br>To examine psychotropic prescription patterns in adult Asian SZ patients based on Research on Asian Psychotropic Prescription 2016 survey	N = 3537<br>Male 59.7%<br>Age 38.7 ± 11.6 years	31.3% FGA<br>80.8% SGA<br><br>42.6% APP<br><br>Out of entire cohort:<br>6.3% FGA + FGA<br>15.7% FGA + SGA<br>20.5% SGA + SGA<br><br>36% risperidone<br>20.1% olanzapine<br>18.9% CLZ<br><br>Mean AP dose: 424 ± 376mg/day CPZeq<br><br>About 50% low-dose AP (<300mg/day CPZeq)<br><br>33% medium dose AP (300-599mg/day CPZeq)<br><br>4.7% high-dose AP (>1200mg/day CPZeq)	13.7% on adjunctive MS	11.7% on adjunctive AD	27.8% on adjunctive BZD<br>45.6% on adjunctive anticholinergics<br><br>High-dose AP use (>1000mg/day CPZeq) a/w:<br>Male<br>↑ Severity of positive and negative symptoms<br>Inpatient treatment<br>↑ Frequency of FGA, SGA, APP, and anticholinergic prescriptions

22	Sultana et al / 2019 / Italy, Spain, United Kindom, United States [22]	Register-based retrospective cohort study<br><br>To describe AP utilization patterns among patients with SZ & SZA disorder in Italy, Spain, the UK, and the USA	N = 765573 new AP treatment episodes	(all these % here are out of all new AP treatment episodes)<br><br>SGA use:<br>86% - 87% US<br>76.9% Spain<br>68.8% Italy<br>64.4% UK<br><br>LAI<br>21.2% UK<br>13.8% Spain<br>9.9% Italy<br>2.4% - 4.2% US<br><br>APP<br>14.8% Spain<br>2.3% UK<br>3.4% Italy<br>3.7% - 5.6% US<br><br>Use of LAI more common among FGA than SGA<br><br>Persistence to AP treatment at 1 year is low (45% in Spain to 30% in Italy)	Adjunctive anticonvulsant use:<br>28.7% - 30.8% US<br>30.4% Italy<br>17.5% Spain<br>8.4% UK<br><br>Adjunctive lithium use:<br>4.2% Italy<br>2.6% Spain<br>3.8% UK<br>4.6% - 6.6% US	Adjunctive AD use: Ranged from 22.8% in UK to 30.5% in Italy in Europe<br><br>52.9% - 54.9% US	

23	Stroup et al / 2019 / United States [23]	Retrospective cohort study<br><br>To study the comparative real-world effectiveness of adjunctive psychotropic treatments for patients with SZ/SZA/SZPH	N = 81921<br>Male 54.2%<br>Age 40.7 ± 12.4 years<br><br>Inclusion:<br>On single SGA (excluding CLZ) + initiated use of a new psychotropic medication during 2001 to 2010	31.8% adjunctive AP	15.7% adjunctive MS	38.0% adjunctive AD	Compared with patients initiating a new adjunctive AP…<br><br>Initiating AD a/w:<br>↓ Risk of psychiatric hospitalisation and psychiatric ED visits<br><br>Initiating BZDs a/w:<br>↑ Risk of hospitalisation, psychiatric ED visits, mortality<br><br>Initiating MSs a/w:<br>↑ Risk of mortality (mainly from gabapentin)<br><br>14.6% adjunctive BZD
24	Qiu et al / 2018 / China and Japan [24]	Register-based retrospective cohort study<br><br>Utilised three large electronic healthcare databases to estimate the prevalence and incidence rates of APP in SZ treatment in China and Japan	China:<br>N = 11961<br>Male 43.1%<br>Age:<br>38.6 ± 17.4 years (mental hospital)<br>28.6 ± 12.5 years (general hospital)<br><br>Japan<br>N = 25034<br>Male 46.6%<br>Age 39 ± 13.5 years	China:<br>87.3% AP monotherapy (78.9% SGA oral, 5.7% FGA oral)<br><br>12.7% APP<br>(12.3% on 2 APs,<br>7.9% SGA + SGA,<br>2.7% SGA + FGA)<br>0.3% on ≥3 APs)<br>(% are out of entire cohort)<br><br>APP fell from 14% in 2010 to 12.8% in 2014<br><br>2.8% LAI monotherapy<br><br>Japan:<br>80.1% AP monotherapy (65.8% SGA oral, 13.2% FGA oral)	Adjunctive MS in China:<br>10.8%<br><br>Adjunctive MS in Japan:<br>24.3%	Adjunctive AD in China:<br>27.4%<br><br>Adjunctive AD in Japan:<br>42.3%	Adjunctive antianxiety medications in China: 31.6%<br><br>Adjunctive antianxiety medications in Japan: 49.9%<br><br>Switching from AP monotherapy to APP was most likely to occur in younger patients with SZ

				19.9% APP (14.6% on 2 AP,<br>6.4% SGA + SGA,<br>5.8% SGA + FGA)<br>5.3% on ≥3 APs<br><br>APP fell from 25.1% in 2010 to 12.3% in 2014<br><br>1.1% LAI monotherapy			
25	Stip & Lachaine / 2018 / Canada [25]	Claim-based cohort study<br><br>To evaluate the impact of early initiation of LAI medications on healthcare resource utilization in patients with SZ/SZA	N = 1996<br>Male 65.8%<br>Age 43.4 ± 14.5 years	48.9% on FGA LAI<br>51.1% on SGA LAI<br><br>38.8% on risperidone LAI<br>17.8% on zuclopenthixol LAI<br>17.7% on fluphenazine LAI<br><br>18.6% CLZ + LAI<br><br>61.9% on LAI + adjunctive oral AP			Use of LAI resulted in (at 1 year follow-up):<br>↓ Hospitalizations<br>↓ Duration of hospitalization<br>↓ ICU visits<br>↓ Duration of ICU stay<br>↓ ES visits<br>↓ Psychiatric department visits<br>↓ Use of all healthcare services<br>↓ Total healthcare costs<br><br>SGA LAI (vs. FGA LAI) users:<br>↓ Age (below 40 years old)<br>Prescribed by psychiatrist<br>↓ Use of oral adjunctive AP at 1-year follow up<br>↓ Duration of hospitalization<br>↑ Medical cost savings

26	Fontanella et al / 2018 / United States [26]	Retrospective cohort study<br><br>To examine trends in long-term APP among Ohio Medicaid patients with SZ/SZA and predictors of use	N = 25062 unique individuals<br>(n = 9211 in 2008, n = 11500 in 2014) with 70,993 observations<br><br>APP defined as being on ≥2 AP for ≥90 days	APP fell (29.5% in 2008 to 24.9% in 2014)<br><br>Out of entire 2014 cohort (n = 11500):<br>21.9% on 2 APs<br>12.5% on SGA + SGA<br>0.44% on FGA + FGA<br>8.4% on FGA + SGA<br>2.1% on CLZ+ SGA<br>0.97% on CLZ + FGA<br><br>1.9% on 3 AP<br>1% on ≥4 AP			APP a/w:<br>↓ Age<br>Whites (vs. African-Americans and minority groups)<br>Male<br>Disabled status<br>Rural residence<br>↑ Medical comorbidities<br>↑ Classes of psychotropics<br>↑ Outpatient / ES visits<br>↓ Inpatient visits<br>No substance use disorder
27	Gaudiano et al / 2018 /United States [27]	To study complex polypharmacy (≥4 psychotropics) pattern in SZ-spectrum (SZA/SZPH/BPD) disorder preadmission	N = 829<br>Male 61.5%<br>Age 39.5 ± 13.5 years<br>White 78.4%<br><br>58.3% schizophrenia/ schizoaffective<br>40.3% psychotic disorder NOS	20.4% (≥4 psychotropics/complex polypharmacy), out of which 36% were APP (i.e 7.4% of entire sample)<br><br>37.5% (≥3 psychotropics/simple polypharmacy)<br><br>57.3% (≥2 psychotropics)<br><br>4.5% on CLZ			Complex polypharmacy with AP/psychotropics a/w:<br>White<br>Disabled<br>Comorbid anxiety disorder<br>↑ Medical comorbidities<br>Tobacco use<br><br>25% on BZD

28	Brostedt et al / 2017 / Sweden [28]	Cross-sectional register-based study<br><br>To compare SZ vs. non-affective psychosis, in terms of use of health care, medical treatment and comorbidity	SZ group:<br>N = 7284<br>Male 57%<br>Age 52.7 ± 14.4 years<br><br>Non-affective psychosis group:<br>N = 11485<br>Male 49%<br>Age 49.5 ± 18.5 years	SZ group:<br>55.5% Oral AP only<br>10.3% LAI only<br>12.5% Oral AP + LAI<br>21.6% No AP<br><br>Around 69% APP<br><br>21.4% olanzapine<br>14.1% zuclopenthixol<br>12.9% risperidone<br>11.6% CLZ<br><br>Non-affective psychosis group:<br>41.9% Oral AP only<br>2.1% LAI only<br>2.9% Oral AP + LAI<br>53.1% No AP<br><br>Around 50% APP<br><br>16.4% olanzapine<br>9.9% risperidone<br>8.2% quetiapine<br>1.2% CLZ	-	SZ group:<br>Around 25%<br><br>Non-affective psychosis group:<br>Around 33%	SZ group anxiolytic use:<br>Around 30%<br><br>Non-affective psychosis group anxiolytic use:<br>Around 28%
29	Tang et al / 2017 / United States [29]	Examined CLZ prescription, APP across providers, and factors associated with these practices (includes those with SZ/SZA)	2010:<br>645 prescribers<br>14072 patients<br><br>2011:<br>632 prescribers<br>13606 patients<br><br>2012:<br>650 prescribers<br>13559 patients	CLZ use was 7% ± 10% of patients per prescriber (range 0% - 89%)<br><br>APP was 7% ± 9% of patients per prescriber (range 0% - 45%)			APP less likely in prescribers with:<br>Caseloads of very ill patients<br>Larger % of Hispanic and black patients<br>Small % of patients with disabilities<br>Larger % of patients with anxiety disorders<br>Low volume of AP prescriptions<br><br>CLZ prescription less likely in prescribers with:

							Larger % of Hispanic and black patients<br>Small % of patients with disabilities<br>Low volume of AP prescriptions<br>Larger % of patients with affective disorders/substance use disorders<br><br>Positive correlation between % of patients with APP and % of patients on CLZ
30	Igbinomwanhia et al / 2017 / Nigeria [30]	Cross-sectional study<br><br>Aimed to determine the prevalence, pattern and correlates of APP among outpatients with SZ	N = 250<br>Male 59.2%<br>Age 37.9 ± 10.6 years<br><br>Illness and AP treatment duration > 1 year for all participants	APP 70.4% (most common was FGA LAI + FGA oral 44.4%)<br><br>29.6% AP monotherapy<br>13.6% oral SGA monotherapy<br>15.2% oral FGA monotherapy<br>1.2% FGA LAI monotherapy<br><br>12% on combination of oral FGAs<br>44% on FGA LAI + oral FGA<br><br>58% on LAI<br><br>Most commonly used FGA LAI was fluphenazine decanoate (52.4% of total sample)<br><br>27.6% on SGA<br><br>Risperidone most commonly prescribed SGA			APP a/w:<br>↑ CPZeq dose<br>↑ Negative symptoms<br>↓ Functioning<br>↑ Side effect burden<br><br>Patients on ≤500mg CPZ-equivalent doses of AP were twice as likely to be on one AP compared with patients on multiple AP<br><br>Patients on polypharmacy with LAI were more likely to be on doses above the British National Formulary limit compared to those on oral-only APP

31	Heald et al / 2017 / United Kingdom [31]	Longitudinal study done between 2004 and 2012<br><br>To determine the change in the number of psychotropic medications prescribed to patients with SZ/SZ-related conditions and psychosis over time, and trends in weight and fasting blood glucose in primary care	N = 195<br><br>LAI AP and CLZ were excluded	Olanzapine was most common AP prescribed in 2012	12% decrease in lithium prescriptions	Significant increase in adjunctive AD over time (1.2% to 5.4%)	
32	Li et al / 2017 / China [32]	Cross-sectional study<br><br>To examined the pattern of adjunctive AD use in SZ and its demographic and clinical correlates in a nationwide survey in China from 2002 to 2012	N = 14013<br>(n = 4486 in 2002<br>n = 5288 in 2006,<br>n = 4239 in 2012)<br>Male 52.9%<br>Age 35.6 ± 13.1 years	40.1% on FGA<br>75.8% on SGA<br><br>28.9% APP<br><br>Mean AP dose: 414.7 ± 309.3 mg/day CPZeq	-	5.2% adjunctive AD in pooled sample<br><br>4.6% in 2002<br>4.3% in 2006<br>6.9% in 2012<br><br>Most commonly prescribed ADs:<br>1.4% Clomipramine in 2002<br><br>1.4% fluoxetine in 2006<br><br>2.1% sertraline in 2012	19.6% on BZD<br><br>Adjunctive AD use a/w:<br>Outpatients in tertiary centres<br>Earlier age of onset<br>↓ Severity of illness<br>↑ Severity of depressive symptoms<br>↓ Likelihood of receiving FGA<br>↑ Likelihood of receiving BZD

33	Fond et al / 2016 / France [33]	Cross-sectional study<br><br>To determine if psychotropics use (SGA vs. FGA, MS, AD, BZD) had association with lower aggression score in SZ/SZA	N = 331<br>Male 75.5%<br>Age 32.5 ± 9.8 years	87% on SGA<br>12.7% on LAI<br>16.3% on CLZ	16.6% on adjunctive MS	27.2% on adjunctive AD	28.1% on adjunctive BZD<br><br>Aggression a/w:<br>↓ Age<br>↓ Education level<br>↑ General psychopathology<br>No SGA use<br>Adjunctive BZD use
34	Ortiz et al / 2016 / United States [34]	Cross-sectional study<br><br>To explore AP prescription practice in adults discharged from state inpatient hospitals	N = 34476 with SZ	11.5% No AP<br>65.1% AP monotherapy<br>23.4% APP			
35	Tesfaye et al / 2016 / Ethiopia [35]	Cross-sectional study<br><br>To examine APP in outpatients with SZ in 2014	N = 412<br>Male 69.7%<br>Age 35.3 ± 10.4 years	28.2% APP<br>60.7% FGA monotherapy<br>11.2% SGA monotherapy<br><br>23.3% FGA + FGA<br>4.6% FGA + SGA<br>0.2% FGA + FGA + SGA			APP a/w:<br>↑ EPSE<br>↑ Number of admissions<br>Substance use<br>↑ Duration of treatment<br>↑ Medication non-adherence<br><br>Patients on treatment for >10 years were about two times more likely to be on APP vs. those on treatment for <5 years<br><br>Patients who had ≥2 previous admissions were three times more likely to be on APP vs. those with no previous admission

36	Tiihonen et al / 2016 / Sweden [36]	Register-based cohort study<br><br>Examine relationship between risk of death and cumulative AP/AD/BZD exposure in SZ	N = 21492<br>Male 61%<br>Age 45.5 ± 11.1 years	90.3% on AP	-	7.6% on adjunctive AD	Moderate and high-dose AP/AD use were associated with 15%–40% lower mortality<br><br>Chronic high-dose use of BZD was associated with up to a 70% higher risk of death compared with no exposure for those on a moderate dose of AP<br><br>2.9% on adjunctive BZD (with AP only)<br>3.1% on AP + AD + BZD
37	Gaviria et al / 2015 / Spain [37]	Observational retrospective study<br><br>To determine the pattern of long-term AP prescription in outpatients with SZ in the province of Tarragona, Spain	N = 1765<br>Male 69%<br>Age 43.6 ± 13.6 years	28.6% AP monotherapy<br>69.6% APP<br>1.8% not on AP<br><br>79.3% on SGA<br>26.2% risperidone<br>14.1% olanzapine<br>12.7% paliperidone<br>5.7% CLZ<br><br>30.1% on 2 AP<br>19.1% on 3 AP<br>10.1% on 4 AP<br><br>44.7% LAI<br>11.4% zuclopenthixol<br>5.4% fluphenazine	26.8% on adjunctive antiepileptics	30% on adjunctive AD	APP a/w:<br>Use of quetiapine/paliperidone as principal treatment<br>Use of LAI<br>↓ Age<br>↑ Concomitant psychotropic use<br>↓ Use of risperidone as principal treatment<br><br>40% on adjunctive anxiolytics<br>26.5% on adjunctive hypnotics/sedatives<br>25.4% on adjunctive anticholinergics

38	Sneider et al / 2015 / Denmark [38]	The national Danish health registers were accessed to examine APP in AZ patients as well as its prevalence and correlates	N = 26000	APP:<br>17.2% in 1996 30.8% in 2006 24.7% in 2012<br><br>19.0% on 2 AP<br>4.8% on 3 AP<br>0.74% on ≥4 AP<br><br>6% on CLZ + APP<br><br>8.5% on LAI + APP<br><br>Out of patients on APP only (n = 6398):<br>FGA + FGA (77.3% in 1996 to 12.0% in 2012)<br><br>FGA + SGA (30.6% in 1996 to 49.3% in 2012)<br><br>SGA + SGA (1.7% in 1996 to 59.2% in 2012)			APP a/w:<br>↑ Number of patients per psychiatrist<br>Females<br>↓ Age<br>Living alone<br>Being institutionalized<br>Receiving early retirement pension<br>↑ Physical comorbidities<br>↑ AP DDD<br>Use of CLZ<br>Use of AD<br>Use of LAI<br>Use of anticholinergics
39	Nasrallah et al / 2015 / United States [39]	The Management of Schizophrenia in Clinical Practice (MOSAIC) Registry was examined to understand burden of SZ	N = 550<br>Male 65.8%<br>Age 42.9 ± 12.9 years<br><br>62% schizophrenia, the rest were schizophreniform or schizoaffective disorder	13.2% haloperidol<br>17.8% risperidone<br>16.5% CLZ<br>14.2% olanzapine			

40	Li et al / 2015 / China [40]	Cross-sectional study<br><br>Examined the use, demographic and clinical correlates of APP and its associations with treatment satisfaction and quality of life in SZ patients in China	N = 4239<br>Male 53.3%<br>Age 36.3 ± 12.8 years	APP:<br>26.1% in 2002 26.4% in 2006<br>34.2% in 2012<br><br>31.3% on 2 AP<br>2.9% on 3 AP<br>0.12% on ≥4 AP<br><br>SGA + SGA increased over time (3.2% in 2002 to 19.2% in 2012)<br><br>FGA + FGA decreased over time (6.0% in 2002 to 0.9% in 2012)<br><br>FGA + SGA decreased over time (16.8% in 2002 to 14.0% in 2012)<br><br>32% on high-dose AP (≥600mg CPZeq)<br><br>20.9% on FGA<br>93.1% on SGA<br><br>Mean AP dose: 492 ± 324mg/day CPZeq<br><br>41.3% risperidone<br>26.4% CLZ<br>15.1% olanzapine			APP a/w:<br>↓ Age of onset<br>↑ Side effects<br>High AP dose (≥600mg/day CPZ-equivalents)<br>On FGA<br>↓ Likelihood of being on BZD<br>↓ Satisfaction with treatment by patients and relatives<br>↑ Mental quality of life<br><br>21.8% on BZD

41	Fisher et al / 2014 / United States [41]	Retrospective claims-based analysis<br><br>To characterize real-world treatment patterns in the prescription of APP compared with AP monotherapy for patients with SZ	N = 4156<br>Male 53.1%<br>Age 40 ± 13.7 years	23.3% APP<br>76.7% AP monotherapy<br><br>18.4% risperidone monotherapy<br>12.9% aripiprazole monotherapy<br>12.2% olanzapine monotherapy<br>4.8% CLZ monotherapy<br>10.3% FGA monotherapy	25.8% on adjunctive MS	48.5% on adjunctive AD	22.3% on adjunctive anti-anxiety medications<br><br>Within one year, 77% of the polypharmacy group and 54% of the monotherapy group discontinued treatment<br><br>Average duration of therapy was 163 ±143 days in the polypharmacy group vs 253 ±147 days in the monotherapy group<br><br>Age < 25 years and APP were independent predictors of treatment discontinuation prior to one year<br><br>↑ Treatment duration a/w:<br>↑ Age<br>AP monotherapy<br>↓ Psychiatric comorbidities<br><br>Characteristics of APP (vs. AP monotherapy):<br>↓ Age<br>↑ Use of adjunctive psychotropics (AD, sedatives, MS, anxiolytics) other than AP<br>↓ Use of anti-hypertensive agents<br>↑ Use of anti-Parkinson agents<br>↑ Treatment discontinuation before 1 year<br>↓ Duration of treatment

42	Xue et al / 2014 / China [42]	Cross-sectional study to evaluate the prescription patterns of AP for inpatients with SZ in China in 2010 and to identify factors influencing these patterns	N = 5251	63.9% SGA<br>44% CLZ<br><br>29% SGA monotherapy<br><br>13.6% SGA + FGA<br><br>13.7% CLZ monotherapy			SGA use a/w:<br>↓ Duration of hospitalization<br>↓ Age<br>Study site
43	Roh et al / 2014 / Korea [43]	Cross-sectional study<br><br>To compare medication of SZ patients discharged from a university psychiatric hospital between 2005 and 2010	N = 194 in 2005<br>Male 43%<br>Age 35.3 ± 12.4 years<br><br>N = 201 in 2010<br>Male 36.8%<br>Age 36.1 ± 11.2 years	APP:<br>37.1% in 2005 to 48.3% in 2010<br><br>Out of entire cohort in 2010 (n = 201):<br>42.3% on 2 AP<br>5% on 3 AP<br>1% on 4 AP<br><br>FGA + FGA (1.5% in 2005 to 0.5% in 2010)<br><br>FGA + SGA (28.9% in 2005 to 24.9% in 2010)<br><br>SGA + SGA (6.7% in 2005 to 23.4% in 2010)<br><br>FGA 34.1% in 2005 to 39.9% in 2010<br><br>Rate of aripiprazole prescription decreased (14.9% in 2005 to 7.0% in 2010)	5.7% in 2005 to 20.9% in 2010	8.2% in 2005 to 8.5% in 2010	APP a/w:<br>High-dose AP (PDD/DDD > 1.5)<br>Hospital stays >30 days<br><br>BZD use: 61.8% in 2005 to 58.7% in 2010<br>Anticholinergic use: 51.5% in 2005 to 56.7% in 2010

				Rate of quetiapine prescription increased (6.2% in 2005 to 27.4% in 2010)<br><br>Most commonly used SGA in 2010<br>33% risperidone<br>27.4% quetiapine<br>23.4% olanzapine<br><br>High-dose AP (PDD/DDD > 1.5) in monotherapy fell from 30.4% in 2005 to 18.4% in 2010<br><br>High-dose APP increased from 34% in 2005 to 45.3% in 2010<br><br>PDD:DDD (2.03 ± 1.05 in 2005 to<br>2.63 ± 5.73 in 2010)			

44	Xiang et al / 2013 / Asia [44]	Cross-sectional study<br><br>To examine trends in the use of AD and their demographic and clinical correlates in the treatment of SZ in Asia between 2001 and 2009	2001:<br>N = 2399<br>Male 55.9%<br>Age 43.6 ± 13.5 years<br><br>2004:<br>N = 2136<br>Male 57.3%<br>Age 43.1 ± 14.2 years<br><br>2009:<br>N = 2226<br>Male 60.6%<br>Age 43.9 ± 13.7 years	Mean AP dose (CPZeq)<br>2001: 641 ± 611mg/d<br>2004: 568 ± 499mg/d<br>2009: 559 ± 447mg/d<br><br>FGA (67.8% in 2001 to 51.9% in 2004 to 41.7% in 2009)<br><br>SGA (45.5% in 2001 to 64.7% in 2004 to 73.7% in 2009)<br><br>APP (46.8% in 2001 to 38.3% in 2004 to 43.4% in 2009)	-	Adjunctive AD:<br>6.8% Pooled overall<br><br>5.3% in 2001<br>6.5% in 2004<br>8.7% in 2009	Adjunctive AD a/w:<br>↓ Age<br>↑ Likelihood of receiving BZD<br>↑ EPSE<br>↓ Positive symptoms
45	Banerjee et al / 2013 / Nepal [45]	Cross-sectional study<br><br>To find the commonest AP prescribed in a tertiary care centre among hospitalized SZ patients in Western Nepal	N = 219<br>Male 61.9%<br>Age (years)<br><40 78.6%<br>>40 21.4%	59% SGA<br>28.6% FGA<br>12.4% FGA + SGA<br><br>34.3% olanzapine<br>28.6% haloperidol<br>17.4% risperidone<br>3.8% CLZ	-	-	

46	Gören et al / 2013 / United States [46]	Retrospective cohort study<br><br>To identify and describe pathways for AP prescribing among veterans with SZ or schizoaffective disorder	N = 1003 into analysis	74% AP monotherapy without CLZ<br><br>19% APP without CLZ<br><br>CLZ utilization was low 0%–2%			Characteristics of APP group (vs. AP monotherapy):<br>↑ Hyperlipidemia<br>↑ Female<br>↑ Caucasian
47	Wu et al / 2012 / Taiwan [47]	Aimed to examine trends in the first-year pharmacological treatment of newly treated SZ patients in Taiwan between 1999 and 2006	N = 2895<br>Male 52.9%<br>Age 36.5 ± 15.1 years	SGA increased (27.5% in 1999 to 76.9% in 2006)<br><br>FGA fell (94.0% in 1999 to 71.3% in 2006)<br><br>LAI fell (17.9% in 1999 to 15.3% in 2006)<br><br>AP monotherapy (44% in 2006 to 36.6% in 2012)<br><br>APP (11% in 1999 to 8.3% in 2012)	Adjunctive MS (13.6% in 1999 to 19.4% in 2006)	Adjunctive AD (37.3% in 1999 to 47.7% in 2006)	Adjunctive anticholinergics (79.9% in 1999 to 58.8% in 2006)<br><br>Adjunctive BZD (89.2% in 1999 to 89.4% in 2006)
48	Himelhoch et al / 2012 / United States [48]	To examine AD use in veterans with SZ in FY 2007	N = 2412<br>Male 92.4%<br>Age 54			Prevalence 37.4%<br><br>Incidence 14.7%<br><br>84.5% SSRI/SNRI<br>22.9% TCA<br>16.1% non-SSRI, non-TCA	AD use a/w:<br>Comorbid depression, PTSD, substance use disorder<br>No history of homelessness<br>Receiving care in specialty outpatient mental health clinics

						77.8% on 1 AD<br>20.8% on 2 AD<br>1.4% on 3 AD	
49	Xiang et al / 2012 / Asia [49]	Cross-sectional study<br><br>Trend and correlates of APP in SZ patients in Asia from 2001 to 2009	N = 6441 (2399 in 2001, 2136 in 2004, 2226 in 2009)<br><br>55.9% males in 2001<br>57.3% males in 2004<br>60.6% males in 2009<br><br><br>43.6 ± 13.5 years (2001)<br>43.1 ± 14.2 years (2004)<br>43.9 ± 13.7 years (2009)	APP<br>46.8% in 2001 38.3% in 2004 43.4% in 2009<br><br>FGA<br>67.8% in 2001<br>51.9% in 2004 41.7% in 2009<br><br>SGA<br>45.5% in 2001 64.7% in 2004 73.7% in 2009<br><br>FGA + FGA 26.5% in 2001 12.7% in 2004 7.8% in 2009<br><br>SGA + SGA<br>3.9% in 2001 5.5% in 2004 13.8% in 2009<br><br>FGA + SGA 16.3% in 2001 20.1% in 2004 21.2% in 2009<br><br><br>In 2001:<br>1.3% on 0 AP<br>51.9% on 1 AP			APP a/w:<br>↓ Age<br>↑ CPZeq dose of AP<br>↑ Severity of positive and negative symptoms<br>↑ Likelihood of receiving LAI and FGA

				33.3% on 2 AP<br>13.5% on ≥3 AP<br><br>In 2004:<br>1.7% on 0 AP<br>60.0% on 1 AP<br>29.0% on 2 AP<br>9.3% on ≥3 AP<br><br>In 2009:<br>2.7% on 0 AP<br>54.4% on 1 AP<br>32.8% on 2 AP<br>10.1% on ≥3 AP<br><br>Risperidone 19.7% in 2001 to 31.5% in 2009<br><br>Haloperidol<br>28.8% in 2001 to 17.9% in 2009<br><br>CLZ<br>14.5% in 2001 to 15.6% in 2009			
50	Mundt et al / 2012 / Uzbekistan vs Germany [50]	Cross-sectional study<br><br>Comparison of AP prescription in inpatients with SZ on one day in Oct 2008	Tashkent<br>N = 503<br>Male 54%<br>Age 43 ± 13 years<br><br>German<br>N = 269<br>Male 58%<br>Age 41	Tashkent:<br>96% on AP<br>67% APP<br>66% CLZ<br>62% haloperidol 13% chlorpromazine<br><br>Average CLZ dose (69 ± 51mg/day)<br><br>Germany:<br>94% on AP<br>44% APP	Tashkent:<br>16% on adjunctive antiepileptic<br><br>Germany:<br>13% on adjunctive antiepileptic	Tashkent:<br>36% on adjunctive AD<br><br>Germany:<br>14% on adjunctive AD	Germany:<br>37% on BZD

				21% olanzapine<br>20% CLZ<br>17% quetiapine<br>17% risperidone<br><br>Average dose of CLZ (392 ± 215mg/day)			
51	Tsutsumi et al / 2011 / Japan [51]	Cohort study<br><br>To examine the evolution of AP switch and polypharmacy in patients with SZ 2 years after first visit	N = 300<br>Male 42%<br>Age 38.5 ± 15.1 years	33% on 0 AP<br><br>43.7% APP<br><br>208 patients started with AP monotherapy<br><br>34.1% of the patients gave up AP monotherapy to move to AP switch (27.4%) and/or APP (17.8%) within 2 years			The main reason for AP switch was 'ineffectiveness'; interestingly, this happened despite the fact that the monotherapy dose was below the recommended range in 47.4% of the AP switches<br><br>The main reason for increase in number of AP prescribed was ‘ineffectiveness’
52	Sim et al / 2011 / Asia [52]	Cross-sectional study<br><br>Examined frequency of MS use and its clinical correlates among hospitalized Asian patients with SZ in 2001–2008	N = 6761<br>(n = 2399 in 2001<br>n = 2136 in 2004<br>n = 1906 in 2008)<br>Male 57.8% (overall)<br>Age 43.5 ± 13.8 years (overall)	Mean AP dose (CPZeq) fell:<br>633 ± 616mg/day (2001)<br>558 ± 505mg/day (2004)<br>559 ± 458 mg/day (2008)<br>580 ± 534mg/day (overall)<br><br>APP fell over time:<br>46.8% (2001)<br>38.3% (2004)<br>42.9% (2008)<br><br>FGA fell over time:	Adjunctive MS increased:<br>20.2% (2001)<br>19.5% (2004)<br>23.7% (2008)<br>20.4% (overall)<br><br>Adjunctive valproate increased:<br>7.6% (2001)<br>10.9% (2004)<br>16.7% (2008)		Adjunctive MS use a/w:<br>↑ Aggression<br>↑ Disorganized speech<br>Year sampled (2008 > earlier years)<br>Multiple hospitalizations<br>↓ Age<br>↓ Negative symptoms<br>Study site

				61.8% (2001)<br>51.9% (2004)<br>40.3% (2008)<br><br>SGA increased over time:<br>45.5% (2001)<br>64.7% (2004)<br>76.6% (2008)<br><br>LAI fell over time:<br>16% (2001)<br>9.7% (2004)<br>10.1% (2008)	11.1% (overall)<br><br>Adjunctive lithium fell:<br>6.8% (2001)<br>5.6% (2004)<br>5.1% (2008)<br>5.6% (overall)		
53	Nielsen et al / 2010 / Denmark [53]	Register-based cohort study<br><br>To investigate treatments and outcomes during the first year after the diagnosis of SZ from 1996 to 2005	N = 13600	Mean AP dose CPZeq increased (93mg/day in 1996 to 232mg/day in 2005)<br><br>FGA use decreased<br><br>SGA use increased (15.3% in 1996 to 89.2% in 2005)<br><br>APP increased (16.7% in 1996 to 37.1% in 2005)<br><br>LAI decreased (22.5% in 1996 to 13.0% in 2005)<br><br>FGA + FGA fell (74.4% in 1996 to 3.1% in 2005)<br><br>SGA + SGA increased (1.7% in 1996 to 41.7% in 2005)<br><br>FGA + SGA increased (23.8% in 1996 to 55.3% in 2005)	Adjunctive MS use no significant change over time	Adjunctive AD increased (24.3% in 1996 to 40.6% in 2005)<br><br>Mean annual DDD increased for AD	Adjunctive anticholinergics fell (38.5% in 1996 to 14.4% in 2005)

				Patients on APP prescribed more than twice the AP dose compared to those on AP monotherapy			
54	Okumura et al / 2010 / Japan [54]	Retrospective cohort study<br><br>To compare the prevalence of diabetes between SZ patients and the general population and to investigate whether diabetes status correlates with the prescription patterns of AP at hospital discharge	N = 3849<br>Male 49.5%<br>Age 45.3 ± 14.3 years	Patients without diabetes (n = 3517):<br>44.8% risperidone<br>27.7% levomepromazine<br>21.4% olanzapine<br><br>Patients with diabetes (n = 332):<br>45.5% risperidone<br>34% levomepromazine<br>33.4% haloperidol<br><br>28.3% FGA<br>31.2% SGA<br>40.6% FGA + SGA			The odds of being prescribed FGA (vs. SGA or FGA + SGA combination) were about 2 times higher among patients with diabetes than without diabetes
55	Kroken et al / 2009 / Norway [55]	Cross-sectional study<br><br>To evaluate the practice of treatment of SZ patients with AP at discharge from acute inpatient settings at a national level	N = 486<br>Male 61.1%<br>Age 40.8 ± 13.5 years	Olanzapine:<br>25.1% at admission<br>29.0% at discharge<br><br>Risperidone:<br>18.7% at admission<br>19.1% at discharge<br><br>Zuclopenthixol:<br>10.5% at admission<br>14.0% at discharge			APP a/w:<br>↓ Age<br>Inpatient treatment in past year<br>Comorbid personality disorder<br>Mental retardation<br><br>FGA prescription at discharge a/w:<br>Inpatient treatment in past year<br><br>Higher CPZeq dose at discharge a/w:<br>↓ Age

				LAI:<br>35.6% at discharge<br><br>CLZ:<br>7.6% at admission<br>8.0% at discharge<br><br>At discharge:<br>7.6% no AP<br>59.5% AP monotherapy (out of which 45.2% were on SGA)<br><br>32.9% APP<br><br>6.0% on ≥2 FGA<br>8.6% on ≥2 SGA<br>18.3% on FGA + SGA<br><br>Mean AP dose: 450 ± 347mg/day CPZeq			Delusions/hallucinations at admission<br>↑ Illness episodes<br>Inpatient treatment during previous year
56	Olfson et al / 2009 / United States [56]	Cross-sectional study<br><br>Compare background characteristics, pharmacologic treatment, and service use of adults treated for schizoaffective disorder and adults treated for SZ	Schizoaffective disorder group:<br>N = 16570<br>Male 46.7%<br><br>SZ group:<br>N = 38760<br>Male 64.2%	Schizoaffective disorder group:<br>87.3% on AP<br><br>SZ group:<br>87.0% on AP	Schizoaffective disorder group:<br>51.6% on adjunctive MS<br><br>SZ group:<br>32.4% on adjunctive MS	Schizoaffective disorder group:<br>56.2% on adjunctive AD<br><br>SZ group:<br>40.6% on adjunctive AD	Patients with schizoaffective disorder<br>were significantly more likely than patients with schizophrenia to receive antidepressants, mood stabilizers, and anxiolytics

57	Sim et al / 2009 / East Asia [57]	Cross-sectional study<br><br>Aim to examine the frequency of high-dose (CPZeq >1000) AP prescriptions in SZ and their clinical correlates in the context of a comparison between studies in 2001 and 2004 within six East Asian countries and territories	In 2001<br>N = 2399<br>Male 55.9%<br>Age 43.6 ± 13.5 years<br><br>In 2004<br>N = 2136<br>Male 57.2%<br>Age 43.1 ± 14.2 years	High-dose AP use fell (17.9% in 2001 to 6.5% in 2004)<br><br>Mean AP dose fell (672.9 ± 645.4mg/day in 2001 to 482.4 ± 413.8mg/day CPZeq in 2004)<br><br>LAI use fell (15.3% in 2001 to 10.4% in 2004)<br><br>FGA use fell (67.8% in 2001 to 53.5% in 2004)<br><br>SGA use rose (45.5% in 2001 to 63% in 2004)<br><br>In 2001:<br>28.8% haloperidol<br>23.4% chlorpromazine<br>19.7% risperidone<br>14.5% CLZ<br><br>In 2004:<br>29.5% risperidone<br>18.1% haloperidol<br>16.3% chlorpromazine<br>15.9% CLZ			High-dose AP a/w:<br>↑ Number of hospital admissions<br>↓ Age<br>↑ Positive symptoms<br>↑ Likelihood of FGA use<br>APP<br>↑ Likelihood of LAI use<br>Study site
58	Citrome et al / 2009 / United States [58]	Retrospective cohort study<br><br>To characterize changes over time in dosing for the initial and subsequent prescriptions of first-line second-	Medicaid population:<br>N = 25658<br>Male 52.4%<br>Age 38.5 years<br><br><br>Commercial population:	Start dose → end dose over 3-month period (CPZeq)<br><br>Medicaid population:<br>Aripiprazole 20.94 → 19.30mg/day<br>Olanzapine 16.07 → 17.88mg/day			

		generation AP used during treatment episodes<br>for outpatients with SZ	N = 2776<br>Male 49.7%<br>Age 40.2 years<br><br>All patients were on at least one oral SGA	Quetiapine 358.83 → 382.56mg/day<br>Risperidone 4.34 → 4.15mg/day<br>Ziprasidone 112.08 → 138.32mg/day<br><br>Commercial population:<br>Aripiprazole 20.20 → 16.99mg/day<br>Olanzapine 11.73 → 13.47mg/day<br>Quetiapine 214.24 → 255.03mg/day<br>Risperidone 3.89 → 3.49mg/day<br>Ziprasidone 107.33 → 125.83mg/day			
59	Xiang et al / 2009 / China [59]	Cross-sectional study<br><br>Examine the frequency and sociodemographic and clinical correlates of MS prescription for outpatients with SZ in Hong Kong and Beijing	N = 505<br>Male 48.1%<br>Age 43.0 ± 8.5 years	17.2% CLZ<br><br>48.3% FGA use only<br><br>Mean AP dose:<br>273 ± 188mg/day CPZeq	6.7% on adjunctive MS<br><br>4.4% sodium valproate 1.6% lithium<br>0.8% carbamazepine		Use of adjunctive MS a/w:<br>↑ Number of admissions<br>Study site<br>Male<br>↓ Duration of illness

60	dosReis et al / 2008 / United States [60]	Cohort study<br><br>To characterize the longitudinal patterns of AP treatment and to investigate the relationship between AP treatment patterns and acute hospitalizations among adults with SZ	Any AP use group:<br>N = 1613<br>Male 50%<br>Age 40 ± 10 years<br><br>No AP use group:<br>N = 114<br>Male 41%<br>Age 41 ± 10 years				Compared to individuals with a more continuous pattern of AP treatment, individuals with moderate or light use had odds of hospitalization for schizophrenia that were 52 or 72% greater<br><br>Light users of AP have an average length of stay per hospitalization that is approximately 20% longer than the average for continuous users
61	Chen et al / 2007 / United States [61]	Retrospective cohort study<br><br>Aim to compare AP with adjunctive MS vs. AP only in treating SZ on outcomes of total health expenditures, inpatient hospitalizations, long-term care stays, ER visits	AP with adjunctive MS<br>N = 1155<br><br>AP without MS<br>N = 7344	-	Out of total sample (n = 8499):<br>13.6% on adjunctive MS<br><br>9.6% valproate<br>1.7% lithium<br>1% carbamazepine<br>1.2% more than one MS	-	Prescription of adjunctive MS a/w:<br>Caucasian<br>↓ Age<br>History of antiepileptic medication use<br>Absence of cardiovascular diagnoses (especially for lithium)<br>↑ Personality disorder diagnosis, drug/alcohol related psychosis, chronic organic psychosis (especially for valproate)<br>↓ Likelihood of anti-Parkinson's medication<br>↑ Pre-index hospitalizations, psychiatrist visits<br>↑ Prior total health expenditure<br><br>Prescription of adjunctive MS not related to:<br>Past utilization of CLZ<br><br>Adjunctive MS recipients had significantly longer AP treatment

							durations than the subjects who did not have exposure to MS<br><br>Those receiving adjunctive valproate were more likely to be receiving SGA as well
62	Xiang et al / 2007 / China [62]	Cross-sectional study<br><br>Examined the frequency and sociodemographic and clinical correlates of APP in SZ patients in Hong Kong and Beijing	N = 398<br>Male 51%<br>Age 42.9 ± 8.5 years	17.6% APP (out of which 45.7% on SGA)<br><br>82.4% AP monotherapy (out of which 47% on SGA)<br><br>22.1% on LAI<br>46.7% on SGA<br>15.6% on CLZ<br>Mean AP dose: 274 ± 192mg/day CPZeq<br><br>Mean AP dose for those on APP:<br>380.3 ± 236.9mg/day CPZeq<br><br>Mean AP dose for those on AP monotherapy:<br>251.9 ± 173.3mg/day CPZeq			APP a/w:<br>↓ Age<br>↑ Number of hospital admissions<br>Study site<br>Use of LAs<br><br>47.7% on anticholinergics

63	Megna et al / 2007 / United States [63]	Cross-sectional study<br><br>To investigate the frequency, effectiveness, and safety of polypharmacy with SGA in patients with SZ or schizoaffective disorder who are chronically hospitalized	N = 26<br>Male 38.5%<br>Age 43.5 ± 11.3 years<br><br>All patients were on put on >1 SGA	At baseline:<br>23.1% on SGA monotherapy<br>38.5% on SGA + FGA<br>19.2% on 2 SGA<br>11.5% on 2 SGA + 1 FGA<br>3.8% on 3 SGA<br><br>At 6 months after addition of at least 1 SGA:<br>57.7% on 2 SGA<br>26.9% on 2 SGA + 1 FGA<br>11.5% on 3 SGA<br>3.8% on 3 SGA + 1 FGA<br><br>AP dose:<br>1386.6 ± 625.1mg/day CPZ-equivalents (at 6 months)<br><br>At baseline:<br>23.1% CLZ<br>26.9% risperidone<br>57.7% olanzapine<br>23.1% haloperidol<br><br>At 6 months:<br>46.2% CLZ<br>69.2% risperidone<br>91.3% olanzapine<br>19.2% haloperidol	26.9% - 34.6% on at least one adjunctive MS	30.8% on at least one adjunctive AD	19.2% - 23.1% on at least one adjunctive BZD<br><br>Use on anticholinergics increased significantly over 6 months

64	Kreyenbuhl et al / 2007 / United States [64]	Cross-sectional study<br><br>Describe patient characteristics and treatment patterns associated with long-term use of APP in veterans with SZ or schizoaffective disorder	APP group:<br><br>N = 5826<br>Male 95%<br>Age 51.3 ± 10.4 years<br><br><br>AP monotherapy group:<br>N = 39745<br>Male 95%<br>Age 53.3 ± 11.9 years	Mean AP dose (mg/day CPZeq):<br><br>APP group:<br>CLZ 507.7<br>Olanzapine 15.8<br>Quetiapine 347.3<br>Risperidone 4.8<br><br>Chlorpromazine 287.8<br>Fluphenazine 768.7<br>Haloperidol 703.4<br><br>Monotherapy group:<br>CLZ 496.9<br>Olanzapine 14.0<br>Quetiapine 309.8<br>Risperidone 4.3<br><br>Chlorpromazine 316.0<br>Fluphenazine 777.9<br>Haloperidol 675.2	Patients receiving APP had a higher likelihood of prescription of adjunctive MS (37%), compared with patients on AP monotherapy (27%)		APP a/w:<br>↓ Age<br>Unmarried<br>Disability<br>Psychiatric hospitalization in the previous year<br>↑ Outpatient mental health visits<br>Caucasian<br>Absence of comorbid depression or substance use disorder<br>↓ Physical comorbidities<br><br>Patients given prescriptions for APP received lower dosages of FGA compared with patients given prescriptions for AP monotherapy<br><br>Patients receiving APP had a higher likelihood of prescription of antianxiety medications (41%), compared with patients on AP monotherapy (33%)<br><br>Patients receiving APP had a higher likelihood of prescription of antiparkinson medications (50%), compared with patients on AP monotherapy (36%)

65	Shi et al / 2007 / United States [65]	Cohort study<br><br>Compare patient characteristics and AP use patterns between individuals with SZ treated in usual care with FGA LAI and those treated with oral AP	LAI group:<br>N = 569<br>Male 67%<br>Age 40.5 ± 10.2 years<br><br>Oral AP group:<br>N = 1617<br>Male 59%<br>Age 42.3 ± 11.4 years	26% (of n = 2186) were treated with FGA LAI at least once during the three-year study, whereas 74% (of n = 2186) were treated with only oral AP and all of this group received FGA			Use of FGA LAI a/w:<br>African-Americans<br>Non-veteran<br>Alcohol or substance use<br>Arrested 6 months prior<br>Inpatient psychiatric admission in previous year<br>↑ Psychotic symptoms and disorganized thinking
66	Mao et al / 2007 / China [66]	Cross-sectional study<br><br>To determine current patterns of AP medication use and metabolic complications among hospitalized Chinese patients with SZ	N = 503<br>Male 50.7%<br>Age<br>41.5 ± 12.3 years (for males)<br>44.7 ± 13.3 years (for females)	90.9% on AP monotherapy<br><br>9.1% on APP/two AP<br><br>0% on more than 2 psychotropics<br><br>30.2% CLZ<br>12.5% risperidone<br>12.2% perphenazine<br><br>2% on LAI<br><br>Mean dosage of CLZ: 298.3 ± 125.8mg/day	4.2% on adjunctive MS	4.4% on adjunctive AD	CLZ use (vs. those on FGA/SGA):<br>Relapse of illness<br>↑ Duration of illness<br>Being overweight as a side effect (vs. SGA)<br>Hyperlipidemia as a side effect (vs. SGA)<br><br>SGA use (vs. those on FGA/CLZ):<br>First-episode schizophrenia<br>↓ Duration of illness<br><br>FGA use (vs. those on SGA/CLZ)<br>Males<br>Being overweight as a side effect (vs. SGA)<br>Hyperlipidemia as a side effect (vs. SGA)

67	Davids et al / 2006 / Germany [67]	Cross-sectional study<br><br>To establish a basis for a monitoring of prescription habits and for pharmacoeconomic considerations in those with SZ, schizotypical, and delusional disorders	In 1998:<br>N = 3984<br>Male 50.6%<br>Age 44.4 years<br><br>In 2001:<br>N = 406<br>Male 54.0%<br>Age 44.1 years<br><br>In 2003:<br>N = 378<br>Male 56.2%<br>Age 41.5 years	FGA and CLZ use fell significantly (CLZ use 18% in 1998 to 9% in 2003)<br><br>SGA use increased significantly<br><br>About 3% - 4% on LAI	Lithium use fell significantly<br><br>Antiepileptic use increased significantly		
68	Hanssens et al / 2006 / Belgium [68]	Cross-sectional study<br><br>Describe the use of AP treatments in ambulatory patients suffering from SZ in Belgium	N = 1000<br>Male 64.1%<br>Age 40.5 ± 11.6 years	1.2% not on AP<br><br>69% SGA (out of which 34.4% on risperidone, 27.4% on olanzapine, 18.9% on CLZ)<br><br>45.6% on FGA<br><br>53.2% SGA monotherapy<br>29.8% FGA monotherapy<br>15.8% FGA + SGA<br><br>21.5% LAI<br><br>73% AP monotherapy (out of which about 50% of patients on quetiapine)<br><br>22.8% on 2 AP<br>3.7% on 3 AP<br>0.5% on 4 AP	15.2% on adjunctive MS	39.1% on adjunctive AD	SGA use associated with less concomitant medication<br><br>21.6% on adjunctive anticholinergics<br><br>Adjunctive anticholinergic use is significantly higher in those on any FGAs<br><br>36.2% on adjunctive BZD<br><br>Higher proportion of those on SGA being treated with monotherapy (93.8%) as compared to those on FGA (74.5%)

				About 20% of patients on higher than recommended AP doses<br><br>Mean CLZ dose:<br>286.4 ± 146.5mg/day			
69	Wheeler et al / 2006 / New Zealand [69]	Cross-sectional study<br><br>Describe AP prescribing practices for<br>outpatients with SZ or schizoaffective disorder over a 3-year period in two large mental<br>health catchment areas of Auckland	N = 6856<br>Male 67%<br>Age 37 years	Oral SGA increased (from 68.7% in 2000 to 87.3% in 2003)<br><br>Oral FGA decreased (from 24.9% in 2000 to 7.4% in 2003)<br><br>FGA LAI decreased (from 23.3% in 2000 to 15.5% in 2003)<br><br>AP monotherapy (81.7% in 2000<br>85.8% in 2001 85.4% in 2003)<br><br>On 2 AP<br>(17.1% in 2000 13.4% in 2001 13.7% in 2003)<br><br>On ≥3 AP<br>(1.2% in 2000 0.8% in 2001 0.9% in 2003)			Those on APP had a higher dose of AP than those on AP monotherapy

				Significant reduction (3.7%) in APP over time<br><br>Overall prescribing of SGAs increased over time<br><br>CLZ use increased (22% to 35%) and was the most prescribed AP in 2003<br><br>Olanzapine use increased (9% to 24%)<br><br>Quetiapine use increased (0.3% to 8.5%)<br><br>Risperidone use fell (38% to 24%)<br><br>High-dose AP (>1000mg CPZeq):<br>3.2% in 2000<br>2.6% in 2001<br>2.2% in 2003<br><br>81.3% of those on high-dose AP were on APP			
70	Kreyenbuhl et al / 2006 / United States [70]	Cross-sectional study<br><br>To identify the persistent use of AP combinations in a nationwide sample of VA patients with SZ and	N = 61257<br>Male 95%<br>Age 52.6 ± 11.7 years	The prevalence of APP was 20.0%, 13.1%, and 9.5% when defined by a ≥30, ≥60, or ≥90-day overlapping supply of APs, respectively<br><br>Out of those on APP for ≥90 days (n = 5826):			Out of those on ≥90 days of APP (n = 5826):<br>Majority of APP with olanzapine (74.8%) or risperidone (65.7%) were used with an FGA

		schizoaffective disorder		97.8% on 2 AP<br>1.3% on 3 AP<br>0.03% on 4 AP<br><br>74.1% on FGA + SGA<br>18.2% on SGA + SGA<br>6.4% on FGA + FGA			
71	Burns et al / 2006 / United Kindom [71]	Cohort study<br><br>To investigate AP prescribing patterns in SZ patients over 2 years	N = 602<br>Male 65%<br>Age 42.7 ± 13.4 years	Prescribing patterns over 2 years:<br><br>FGA only (31.6% to 30.0%)<br><br>FGA (alone or in combination with other AP): 59% to 48%<br><br>SGA only (39.0% to 47.4%)<br><br>SGA (alone or in combination with other AP): 62.5% to 65.7%			Inpatient care remains the main forum for switching of AP<br><br>Medication changes were significantly more frequent in those who were admitted. Overall, the classes of medication were changed in 29–36% of those admitted across the different time periods and only 11–14% of those not admitted in each period<br><br>Change is generally toward use of more SGA only
72	Haro et al / 2006 / Europe [72]	Cross-sectional study<br><br>To describe the correlates of 12-month AP maintenance for SZ in the outpatient setting	N = 7186<br>Male 56.9%<br>Age 40.0 ± 13.2 years	Medication maintenance at 12 months varied with the type of AP prescribed, being highest with CLZ (79.5%) and olanzapine<br>(77.0%), and lowest with quetiapine (51.4%) and amisulpride (58.2%)<br><br>Mean CLZ dose at 12 months: 252.5 ± 144.9mg/day	Adjunctive MS from baseline to 12 months:<br>9.5% to 9.0%	Adjunctive AD from baseline to 12 months:<br>18.8% to 19.0%	Those starting CLZ (vs. those starting other medications):<br>↓ Age of onset<br>↑ Severity of illness<br>↑ Medication maintenance at 12 months<br><br>14.4% to 9% adjunctive anticholinergics from baseline to 12 months<br>35.1% to 26.8% adjunctive anxiolytics/hypnotics from baseline to 12 months

							Discontinuation of AP medication a/w:<br>Alcohol abuse/dependence<br>Use of MS<br>Involuntary admission<br>Arrested 6 months prior<br>↑ Clinical severity<br>Previous AP use before enrolment<br>No social activities 4 weeks before enrolment<br>No loss of libido
73	De Hert et al / 2006 / Belgium [73]	Cross-sectional study<br><br>To evaluate to what degree AP prescribing patterns are in accordance with published treatment recommendations; to gain insight in factors determining guideline adherence or nonadherence in patients with SZ	N = 1215<br>Male 61.1%<br>Age 35.5 ± 12.0 years	69.4% on SGA<br><br>42.3% on SGA only<br><br>19.1% on LAI FGA<br><br>27.8% on FGA only<br><br>27.1% on FGA + SGA<br><br>57.8% on 1 AP<br>30.6% on 2 AP<br>9.4% on 3 AP<br>2.2% on ≥4 AP<br><br>31.2% on high-dose/overdose of FGA<br><br>10.8% on CLZ<br><br>Mean dose of CLZ: 334.3 ± 144.4mg/day			Those with any FGA use (vs. on SGA only):<br>↑ Age<br>↑ Duration of illness<br>↑ Symptom severity<br><br>AP monotherapy more frequent in patients on SGA<br><br>APP a/w:<br>↑ Duration of illness<br>↑ Symptom severity<br><br>High-dose of FGA a/w:<br>↓ Duration of illness<br>↑ Symptom severity<br><br>High-dose of SGA a/w:<br>First-episode patient

74	Novick et al / 2005 / Europe [74]	Observational cohort study<br><br>To evaluate concomitant medication usage of non-AP drugs during the first 6 months of the prospective observational European Outpatient Health Outcomes (SOHO) Study involving patients with SZ on AP	N = 8057 on AP monotherapy	40.4% olanzapine<br><br>20.1% risperidone<br><br>7.6% quetiapine<br><br>3.4% on CLZ<br><br>6.1% on oral FGA<br><br>4.6% on FGA LAI	Frequency of adjunctive MS use at 3 months and 6 months ranged from 6.6% to 19.3% depending on type of AP	Frequency of adjunctive AD use at 3 months and 6 months ranged from 8.3% to 23% depending on type of AP	Frequency of adjunctive anticholinergic use at 3 months and 6 months ranged from 5.3% to 28.9% depending on type of AP<br><br>Frequency of adjunctive anxiolytic/hypnotic use at 3 months and 6 months ranged from 21.7% to 36.9% depending on type of AP<br><br>Adjunctive anxiolytic/hypnotic use fell at 3 and 6-month visits vs. baseline<br><br>Adjunctive anxiolytic/hypnotic use a/w:<br>↑ Age<br>Country<br>↑ Symptom severity<br>↑ Hostility<br>↑ EPSE<br>↑ Loss of libido as side effect<br>Prior use of anxiolytics<br><br>Adjunctive AD use a/w:<br>Female<br>Country<br>↓ Positive and negative symptoms<br>Compliant patients<br>↑ Depressive symptoms<br>↑ Loss of libido as side effect<br>Prior AD and MS use<br><br>Adjunctive MS use a/w:<br>↑ Hostility<br>Country

							Prior AP and MS use<br>↓ Negative symptoms<br>↑ Depressive symptoms<br><br>Adjunctive anticholinergic use a/w:<br>Non-compliant patients<br>Absence of substance abuse<br>Country<br>Prior olanzapine and CLZ use<br>↑ Positive symptoms<br>↑ EPSE<br>Prior anticholinergic use<br><br>Concomitant non-AP medication use a/w (at 6 months follow-up):<br>↑ Incidence of sexual side-effects<br>↑ Extrapyramidal side effects<br><br>A high proportion of patients in the CLZ,<br>olanzapine and risperidone cohorts were still taking the AP started at baseline with no addition of other AP at the 6-month visit (83.9%, 83.1% and 78.3%, respectively)

75	Edlinger et al / 2005 / Austria [75]	Cross-sectional study<br><br>Evaluated whether efforts to promote evidence-based guidelines for the psychopharmacological treatment of patients with SZ have led to measurable changes of treatment practice in the hospital	In 1989:<br>N = 58<br>Male 67.2%<br>Age 33.6 ± 12.1 years<br><br>In 1995:<br>N = 80<br>Male 67.5%<br>Age 33.5 ± 10.5 years<br><br>In 1998:<br>N = 98<br>Male 58.2%<br>Age 37.3 ± 13.0 years<br><br>In 2001:<br>N = 97<br>Male 63.9%<br>Age 37.1 ± 13.1 years	Haloperidol use fell (50% in 1989 to 9.3% in 2001)<br><br>Risperidone use fell (31.3% in 1995 to 8.2% in 2001)<br><br>CLZ use fell (32.8% in 1989 to 22.7% in 2001)<br><br>Olanzapine use increased (15.3% in 1998 to 33.0% in 2001)<br><br>CLZ dose increased from 285.2mg/day in 1989 to 344.5mg/day in 2001<br><br>APP during inpatient stay:<br>6.9% in 1989 to 14.4% in 2001<br><br>LAIs:<br>19% in 1989 to 12.5% in 2001	In 2001, only 3.1% patients received adjunctive valproate	Adjunctive AD:<br>12.1% in 1989 to 16.5% in 2001	Adjunctive BZD:<br>75.9% in 1989 to 89.7% in 2001<br><br>Adjunctive anticholinergics:<br>48.3% in 1989 to 14.4% in 2001

76	Faries et al / 2005 / United States [76]	Retrospective cohort study<br><br>To assess the annual rate and duration of AP monotherapy and APP among SZ patients initiated on commonly used SGA medications	Olanzapine group:<br>N = 405<br>Male 61.7%<br>Age 41.8 ± 10.5 years<br><br>Quetiapine group:<br>N = 115<br>Male 47.8%<br>Age 39.6 ± 10.9 years<br><br>Risperidone group:<br>N = 276<br>Male 54.7%<br>Age 40.4 ± 12.1 years	35.7% AP monotherapy predominantly throughout 1 year period<br><br>26.9% APP predominantly throughout 1 year period<br><br>57.7% had at least one prolonged period of APP (>60 consecutive days)<br><br>Patients averaged 195.5 days on AP monotherapy, 155.7 days on APP, and 13.9 days without AP therapy			Olanzapine-initiated patients were significantly more likely to be on monotherapy with the initiating AP during the 1-year post initiation compared to risperidone or quetiapine<br><br>The number of monotherapy days was significantly greater for olanzapine than quetiapine, but not for olanzapine versus risperidone, or for risperidone versus quetiapine-initiated patients
77	Hodgson et al / 2005 / United Kindom [77]	Cross-sectional study<br><br>To examine the long-term effectiveness of SGA in a naturalistic setting for patients with SZ	CLZ group:<br>N = 44<br>Male 81.8%<br>Age 37.3 ± 1.6 years<br><br>Olanzapine group:<br>N = 152<br>Male 59.9%<br>Age 41.8 ± 1.2 years<br><br>Risperidone group:<br>N = 57	CLZ had longest time to ‘failure’ (switching/stopping treatment), followed by olanzapine then risperidone<br><br>Patients taking risperidone were 1.3 times more likely to discontinue than those taking olanzapine<br><br>Mean doses:<br>CLZ 332.3 ± 150.3mg/day<br><br>Olanzapine 12.1 ± 4.6 mg/day<br><br>Risperidone 4.7 ± 2.4mg/day			Likelihood of treatment discontinuation a/w:<br>Females<br>↓ AD use<br>↓ Age<br><br>Reasons cited for treatment discontinuation:<br>Lack of efficacy<br>Poor compliance

			Male 64.9%<br>Age 39.4 ± 2.0 years	0.8% of patients were on higher than recommended doses of AP			
78	Magliano et al / 2004 / Italy [78]	Cross-sectional study<br><br>Aim to describe the type and doses of psychotropic drugs received by outpatients with SZ in Italy	N = 682	98% on AP<br><br>71% AP monotherapy (out of which 18% were lower than official therapeutic doses, and 5% higher than that)<br><br>29% APP<br>24% on 2 AP<br>5% on ≥3 AP<br><br>25% on LAI AP<br><br>55% FGA only<br>35% SGA only<br>10% FGA + SGA<br><br>32.7% on haloperidol<br>24.9% on risperidone<br>16.6% on haldol decanoate<br>16% on CLZ	8% on adjunctive MS	10% on adjunctive AD	25% on adjunctive anticholinergics<br>47% on adjunctive BZD<br><br>BZD prescription a/w:<br>↑ Number of hospitalizations in past year<br>↑ Anxiety symptoms<br>↑ Anticholinergic use<br>↑ APP<br>↓ Education level<br>Living in areas of high or low population density<br><br>↑ Number of neuroleptics prescribed a/w:<br>↑ Severity of positive symptoms<br>↑ Duration of contact with treatment service<br>Living in areas of low population density

				Mean weighted dose of APs<br>Haloperidol-eq 5.8±5.0 mg			APP (vs. those on AP monotherapy):<br>Not on SGA<br>On BZD<br>On anticholinergics<br>Living in areas with low population density<br><br>LAI prescription (vs. oral AP only):<br>Living in areas of low population density<br>↑ Age<br>↓ Educational level<br>↑ Duration of illness<br>↓ Anxiety/depression symptoms<br>↑ AP dose<br><br>AD use (vs. those without AD):<br>↓ Positive symptoms<br>Unemployment<br>↑ Concomitant psychotropic drugs received<br>Anticholinergic use (vs. those not on anticholinergics):<br>↑ Levels of disability<br>On combination of LAI and oral AP<br><br>CLZ use (vs. those not on CLZ):<br>↑ Number of hospital admissions<br>Previous suicide attempt<br>High educational level<br>↓ Likelihood of receiving BZD, anticholinergics, and different neuroleptics<br><br>SGA prescription (vs. those not on SGA):

							↓ Age<br>Living in high population density areas<br>No attempted suicide<br>↓ Frequent use of anticholinergics and LAI<br><br>Combination of SGA + FGA (vs. those on SGA only or FGA only)<br>↑ Positive symptoms<br>↓ Age<br>↓ Duration of contact with treatment service
79	Ganguly et al / 2004 / United States [79]	Retrospective cohort study<br><br>To determine the prevalence, trends, and factors associated with APP in patients with SZ categorized according to type of AP and duration of use and to contrast usage patterns with published treatment guidelines	N = 31435<br>Male 50%<br>Age 43 ± 14 years	23% APP<br><br>Prevalence of APP highest among those on CLZ (36.2%)<br><br>APP increased from 32% in 1998 to 41% in 2000			APP a/w:<br>Males<br>Aged/blind/disabled<br>Weight loss/malnutrition<br>Comorbid epilepsy/psychiatric disorders<br>On antiparkinsons medication<br>On quetiapine/olanzapine, risperidone<br>On chlorpromazine, thioridazine, thiothixene<br>On CLZ<br>Regular AP use/medication adherence<br>No alcohol abuse<br>Cardiac-related diseases<br>Respiratory diseases<br>Complicated diabetes

80	Sim et al / 2004a / Asia [80]	Cross-sectional study<br><br>To examine the use of high-dose AP/LAI and clinical correlates in SZ patients in East Asia	N = 2399<br>Male 55.9%<br>Age 43.6 ± 13.5 years	17.9% high-dose AP (CPZeq > 1000mg)<br><br>15.3% LAI<br><br>45.7% APP<br><br>40% on SGA	-	-	63.7% on anticholinergics<br><br>High-dose AP a/w:<br>Country<br>↓ Age<br>Delusions<br>Disorganized speech<br>APP<br>On LAI but not SGA
81	Sim et al / 2004b / Asia [81]	Cross-sectional study<br><br>Examine AP prescription for patients with SZ from 6 countries and territories within Asia	N = 2399	45.7% APP			APP a/w:<br>Country<br>↓ Age<br>↑ Duration of illness
82	De las Cuevas et al / 2004 / Spain [82]	Cross-sectional study<br><br>To evaluate the prevalence and correlates of polypharmacy in psychiatric patients	N = 113	38.9% on FGA<br>43.4% on SGA<br>22.1% on LAI<br><br>SGA is most prevalent option	20.3% on anticonvulsants	23.9% on AD	54.8% on BZD
83	Rothbard et al / 2003 / United States [83]	Cross-sectional study<br><br>Examine trends in AP medication use in a treated population of publicly funded patients with SZ between 1991 and 1996	No AP prescription group:<br><br>N = 1060<br>Male 62%<br>Age 38.7 ± 10.9 years	CLZ use increased from 3% in 1991 to 10% in 1996<br><br>SGA use increased from 14% in 1994 to 31% in 1996<br><br>28% on risperidone in 1996		32.5% on adjunctive AD	No AP use (vs. those on AP):<br>Males<br>African-Americans<br>↓ Age<br>↓ Disabled<br>↑ Hospitalization rates<br><br>FGA use (vs. those on SGA/CLZ):<br>African-American

			AP prescription group:<br><br>N = 4080<br>Male 56%<br>Age 41.4 ± 11.0 years	FGA use fell (79% in 1991 to 64% in 1996)<br><br>LAI use fell (20% in 1991 to 15% in 1996)			↑ Age<br><br>SGA use (vs. those on FGA/CLZ):<br>↑ Rates of partial hospitalization care and intensive case management usage<br>↑ Inpatient and emergency room usage<br>↑ Adjunctive AD prescription<br><br>CLZ use (vs. those on FGA/SGA):<br>↑ Intensive care management use<br><br>CLZ users least likely to have interruptions to medication use (vs. SGA or FGA)<br><br>Those on CLZ had the lowest psychiatric hospitalization rates and emergency room use rates (vs. FGA and SGA)<br><br>Switching medication more likely for those on SGA (vs. CLZ and FGA)

84	Loosbrock et al / 2003/ United States [84]	Cross-sectional study<br><br>To describe AP medication treatment patterns and estimate the total costs of care associated with treatment patterns for individuals diagnosed with<br>SZ in usual care settings	N = 2082<br>Male 51%<br>Age 51 ± 14 years	25.7% no AP<br><br>52.3% AP monotherapy<br><br>9.4% on APP			APP and switching of AP medication were associated with significant increases in healthcare cost relative to AP monotherapy
85	dosReis et al / 2002 / United States [85]	Cross-sectional study<br><br>To describe oral<br>AP dosing patterns and psychotropic treatments using computerized Medicaid claims data for individuals who were diagnosed with SZ<br>and received treatment on an outpatient basis during 1991	AP only group:<br><br>N = 782<br>Male 37%<br><br>AP and other psychotropic group<br>N = 1424<br>Male 35%<br><br>Other psychotropic only group<br>N = 828<br>Male 35%<br>39% age 35-49	Mean oral AP dose overall: 467 ± 490mg/day CPZeq (729 ± 586mg/day CPZeq for high-potency agents and 304 ± 328mg/day CPZeq for low-potency agents)<br><br>85% on AP<br><br>Among those on AP + adjunctive psychotropics:<br>97% on APP<br><br>Mean daily doses of high-potency agents were 22% above the maximum recommended daily dose (600mg CPZeq)	Among those on AP + adjunctive psychotropics:<br>13% on adjunctive anticonvulsant<br>12% on adjunctive lithium	Among those on AP + adjunctive psychotropics:<br>32% on adjunctive AD	Among those on AP + adjunctive psychotropics:<br>21% on adjunctive BZD<br><br>↑ AP daily dose a/w:<br>High-potency AP<br>Males<br>↓ Age<br>African-Americans on high-potency AP

86	Ungvari et al / 2002 / Hong Kong [86]	Cross-sectional study<br><br>To monitor trends in the pharmacotherapy of SZ and to identify changes in prescribing practices	In 1996 for acute patients:<br>N = 323<br>Male 50.5%<br>Age 36.8 ± 11.5 years<br><br>In 1996 for chronic patients:<br>N = 634<br>Male 67.0%<br>Age 43.7 ± 11.3 years<br><br>In 1999 for acute patients:<br>N = 356<br>Male 54.5%<br>Age 40 ± 14.3 years<br><br>In 1999 for chronic patients:<br>N = 846<br>Male 65.8%<br>Age 46.5 ± 13.2 years	Mean AP dose fell significantly for both chronic and acute patients<br><br>APP fell significantly among chronic patients<br><br>SGA use increased (5.5% to 19.2% in acute patients, 3.4% to 12.3% in chronic patients)<br><br>In 1999, SGA + FGA use:<br>26.2% of acute patients<br>30% of chronic patients	No significant change in adjunctive lithium use (3.4% in 1996 to 2.7% in 1999)<br><br>No significant change in adjunctive anticonvulsant use (5.7% in 1996 to 6.9% in 1999)	No significant change in adjunctive AD use (5.8% in 1996 to 6.2% in 1999)	Patients on FGA + SGA (vs. those on FGA only):<br>↓ AP dose<br>↓ LAI<br><br>BZD use increased (5.5% in 1996 to 8.6% in 1999)

87	Clark et al / 2002 / United States [87]	Retrospective cohort study<br><br>To examine trends in combination therapy in the treatment of SZ	N = 836<br><br>Male<br>51.2% (SZ group)<br>79.2% (schizoaffective disorder group)<br><br>Age<br>47.7 ± 14.9 years (SZ group)<br>43.6 ± 11.6 years (schizoaffective disorder group)	APP quadrupled, increasing from 5.7% in 1995 to 24.3% in 1999<br><br>FGA + SGA use increased from 15.9% in 1995 to 27.7% in 1999<br><br>≥2 SGA use increased (10% of participants in 1999)<br><br>Decrease in FGA and increase in SGA prescription<br><br>70% were on an SGA by 1999	Adjunctive MS use increased from 17.7% in 1995 to 30.0% in 1999	Adjunctive AD use increased from 18.5% in 1995 to 35.6% in 1999 for those with SZ, and 20.3% in 1995 to 48.5% in 1999 for those with schizoaffective disorder	Anxiolytics/hypnotic use increased from 19.9% in 1995 to 33.5% in 1999
88	Meyer et al / 2002 / United States [88]	Cross-sectional study<br><br>To compare the impact of SGA with that of FGA in those with SZ-spectrum disorder	N = 82<br>Male 57%<br>Age 40.8 ± 8.6 years	72% on SGA<br>28% on FGA			24% on adjunctive anticholinergics<br><br>SGA use (vs. those on FGA):<br>↓ Anticholinergic use<br>↓ Hostility/excitement<br>↓ Cognitive impairment<br>↑ Perceived weight gain

AP = antipsychotic. AD = antidepressant. MS = mood stabilizer. APP = antipsychotic polypharmacy. FGA = first generation antipsychotic. SGA = second generation antipsychotic. LAI = long acting injectable. CLZ = clozapine. BZD = benzodiazepine. CPZ = chlorpromazine. SZ = schizophrenia. SZA = schizoaffective disorder. SZPH = schizophreniform disorder. PDD = prescribed daily dose. DDD = defined daily dose. CPZeq = chlorpromazine-equivalents. Li-eq = lithium equivalents.

References:

1. Tamene, F.B.; Sema, F.D.; Sendekie, A.K. Antipsychotic polypharmacy and associated factors among patients with schizophrenia: Multicenter cross-sectional study in Northwest Ethiopia. *PLoS One* **2023**, *18*, e0290037.
2. Miron, A.A.; Petric, P.S.; Teodorescu, A.; Ifteni, P.; Chele, G.; Szalontay, A.S. Benzodiazepines and Mood Stabilizers in Schizophrenia Patients Treated with Oral versus Long-Acting Injectable Antipsychotics-An Observational Study. *Brain Sci* **2023**, *13*.
3. Luthra, S.; Duggan, L.; Agrawal, A.; Kaur, G.; Luthra, N. Prevalence of High-dose Antipsychotic Prescribing in Schizophrenia: A Clinical Audit in a Regional Queensland Mental Health Service. *Int J Appl Basic Med Res* **2023**, *13*, 70-76.
4. Fulone, I.; Silva, M.T.; Lopes, L.C. Use of atypical antipsychotics in the treatment of schizophrenia in the Brazilian National Health System: a cohort study, 2008-2017. *Epidemiol Serv Saude* **2023**, *32*, e2022556.
5. Fond, G.; Falissard, B.; Nuss, P.; Collin, C.; Duret, S.; Rabbani, M.; De Chefdebien, I.; Tonelli, I.; Llorca, P.M.; Boyer, L. How can we improve the care of patients with schizophrenia in the real-world? A population-based cohort study of 456,003 patients. *Mol Psychiatry* **2023**.
6. Lagreula, J.; de Timary, P.; Elens, L.; Dalleur, O. Antipsychotic polypharmacy and clozapine prescribing patterns: evolution and correlates before and after a psychiatric hospitalisation. *Ther Adv Psychopharmacol* **2022**, *12*, 20451253221112587.
7. Vadiei, N.; El-Ali, J.; Delaune, J.; Wild, C.; Liu, Y.S. Patterns and predictors of oral antipsychotic prescribing in adult patients with schizophrenia. *Explor Res Clin Soc Pharm* **2022**, *6*, 100148.
8. Hori, H.; Yasui-Furukori, N.; Hasegawa, N.; Iga, J.I.; Ochi, S.; Ichihashi, K.; Furihata, R.; Kyo, Y.; Takaesu, Y.; Tsuboi, T.; et al. Prescription of Anticholinergic Drugs in Patients With Schizophrenia: Analysis of Antipsychotic Prescription Patterns and Hospital Characteristics. *Front Psychiatry* **2022**, *13*, 823826.
9. Anozie, I.G.; James, B.O.; Omoaregba, J.O.; Oriji, S.O.; Erohubie, P.O.; Enebe, A.C. Correlates of high-dose antipsychotic prescription amongst outpatients with Schizophrenia in a Nigerian Hospital. *S Afr J Psychiatr* **2022**, *28*, 1791.
10. Pai, N.; Acar, M.; Juneja, P.; Kouhkamari, M.H.; Siva, S.; Mullan, J. Antipsychotic prescribing patterns in Australia: a retrospective analysis. *BMC Psychiatry* **2022**, *22*, 110.
11. Guo, X.; Yu, H.; Wang, H. Antipsychotic patterns in outpatients with schizophrenia in China: A cross sectional study. *Medicine (Baltimore)* **2021**, *100*, e26912.
12. Gamon, V.; Hurtado, I.; Salazar-Fraile, J.; Sanfelix-Gimeno, G. Treatment patterns and appropriateness of antipsychotic prescriptions in patients with schizophrenia. *Sci Rep* **2021**, *11*, 13509.
13. Taipale, H.; Puranen, A.; Mittendorfer-Rutz, E.; Tiihonen, J.; Tanskanen, A.; Cervenka, S.; Lahteenvuo, M. Antipsychotic use among persons with schizophrenia in Sweden and Finland, trends and differences. *Nord J Psychiatry* **2021**, *75*, 315-322.
14. Puranen, A.; Koponen, M.; Tanskanen, A.; Tiihonen, J.; Taipale, H. Use of antidepressants and mood stabilizers in persons with first-episode schizophrenia. *Eur J Clin Pharmacol* **2020**, *76*, 711-718.
15. Hata, T.; Kanazawa, T.; Hamada, T.; Nishihara, M.; Yoneda, H.; Nakajima, M.; Katsumata, T. The 12-year trend report of antipsychotic usage in a nationwide claims database derived from four million people in Japan. *J Psychiatr Res* **2020**, *127*, 28-34.
16. Takahashi, T.; Otsubo, T.; Kunisawa, S.; Sasaki, N.; Imanaka, Y. Factors associated with high-dose antipsychotic prescriptions in outpatients with schizophrenia: An analysis of claims data from a Japanese prefecture. *Neuropsychopharmacol Rep* **2020**, *40*, 224-231.
17. Ichihashi, K.; Hori, H.; Hasegawa, N.; Yasuda, Y.; Yamamoto, T.; Tsuboi, T.; Iwamoto, K.; Kishimoto, T.; Horai, T.; Yamada, H.; et al. Prescription patterns in patients with schizophrenia in Japan: First-quality indicator data from the survey of "Effectiveness of Guidelines for Dissemination and Education in psychiatric treatment (EGUIDE)" project. *Neuropsychopharmacol Rep* **2020**, *40*, 281-286.
18. Lim, W.K.; Chew, Q.H.; He, Y.L.; Si, T.M.; Chiu, F.H.; Xiang, Y.T.; Kato, T.A.; Kanba, S.; Shinfuku, N.; Lee, M.S.; et al. Coprescription of mood stabilizers in schizophrenia, dosing, and clinical correlates: An international study. *Hum Psychopharmacol* **2020**, *35*, 1-7.

19. Shenoy, S.; R, A.; Bhandary, R.P.; Praharaj, S.K. Frequency, reasons, and factors associated with antipsychotic polypharmacy in Schizophrenia: A retrospective chart review in a tertiary hospital in India. *Asian J Psychiatr* **2020**, *51*, 102022.
20. Anozie, I.G.; James, B.O.; Omoaregba, J.O. Antipsychotic prescription and polypharmacy among outpatients with schizophrenia in a Nigerian hospital. *Niger Postgrad Med J* **2020**, *27*, 30-36.
21. Dong, M.; Zeng, L.N.; Zhang, Q.; Yang, S.Y.; Chen, L.Y.; Najoan, E.; Kallivayalil, R.A.; Viboonma, K.; Jamaluddin, R.; Javed, A.; et al. Prescription of antipsychotic and concomitant medications for adult Asian schizophrenia patients: Findings of the 2016 Research on Asian Psychotropic Prescription Patterns (REAP) survey. *Asian J Psychiatr* **2019**, *45*, 74-80.
22. Sultana, J.; Hurtado, I.; Bejarano-Quisoboni, D.; Giorgianni, F.; Huybrechts, K.F.; Lu, Z.; Patorno, E.; Sanfelix-Gimeno, G.; Tari, D.U.; Trifiro, G. Antipsychotic utilization patterns among patients with schizophrenic disorder: a cross-national analysis in four countries. *Eur J Clin Pharmacol* **2019**, *75*, 1005-1015.
23. Stroup, T.S.; Gerhard, T.; Crystal, S.; Huang, C.; Tan, Z.; Wall, M.M.; Mathai, C.; Olfson, M. Comparative Effectiveness of Adjunctive Psychotropic Medications in Patients With Schizophrenia. *JAMA Psychiatry* **2019**, *76*, 508-515.
24. Qiu, H.; He, Y.; Zhang, Y.; He, M.; Liu, J.; Chi, R.; Si, T.; Wang, H.; Dong, W. Antipsychotic polypharmacy in the treatment of schizophrenia in China and Japan. *Aust N Z J Psychiatry* **2018**, *52*, 1202-1212.
25. Stip, E.; Lachaine, J. Real-world effectiveness of long-acting antipsychotic treatments in a nationwide cohort of 3957 patients with schizophrenia, schizoaffective disorder and other diagnoses in Quebec. *Ther Adv Psychopharmacol* **2018**, *8*, 287-301.
26. Fontanella, C.A.; Hiance-Steelesmith, D.L.; Guirgis, H.; Campo, J.V. Trends in and Predictors of Long-Term Antipsychotic Polypharmacy Use Among Ohio Medicaid Patients with Schizophrenia, 2008-2014. *Psychiatr Serv* **2018**, *69*, 1015-1020.
27. Gaudiano, B.A.; Guzman Holst, C.; Morena, A.; Reeves, L.E.; Sydnor, V.J.; Epstein-Lubow, G.; Weinstock, L.M. Complex Polypharmacy in Patients With Schizophrenia-Spectrum Disorders Before a Psychiatric Hospitalization: Prescribing Patterns and Associated Clinical Features. *J Clin Psychopharmacol* **2018**, *38*, 180-187.
28. Brostedt, E.M.; Msghina, M.; Persson, M.; Wettermark, B. Health care use, drug treatment and comorbidity in patients with schizophrenia or non-affective psychosis in Sweden: a cross-sectional study. *BMC Psychiatry* **2017**, *17*, 416.
29. Tang, Y.; Horvitz-Lennon, M.; Gellad, W.F.; Lave, J.R.; Chang, C.H.; Normand, S.L.; Donohue, J.M. Prescribing of Clozapine and Antipsychotic Polypharmacy for Schizophrenia in a Large Medicaid Program. *Psychiatr Serv* **2017**, *68*, 579-586.
30. Igbinomwanhia, N.G.; Olotu, S.O.; James, B.O. Prevalence and correlates of antipsychotic polypharmacy among outpatients with schizophrenia attending a tertiary psychiatric facility in Nigeria. *Ther Adv Psychopharmacol* **2017**, *7*, 3-10.
31. Heald, A.; Livingston, M.; Yung, A.; De Hert, M.A. Prescribing in schizophrenia and psychosis: Increasing polypharmacy over time. *Hum Psychopharmacol* **2017**, *32*.
32. Li, Q.; Su, Y.A.; Xiang, Y.T.; Shu, L.; Yu, X.; Ungvari, G.S.; Ng, C.H.; Chiu, H.F.; Ning, Y.P.; Wang, G.H.; et al. Adjunctive antidepressant use in schizophrenia in China: A national survey (2002-2012). *Hum Psychopharmacol* **2017**, *32*.
33. Fond, G.; Boyer, L.; Favez, M.; Brunel, L.; Aouizerate, B.; Berna, F.; Capdevielle, D.; Chereau, I.; Dorey, J.M.; Dubertret, C.; et al. Medication and aggressiveness in real-world schizophrenia. Results from the FACE-SZ dataset. *Psychopharmacology (Berl)* **2016**, *233*, 571-578.
34. Ortiz, G.; Hollen, V.; Schacht, L. Antipsychotic Medication Prescribing Practices Among Adult Patients Discharged From State Psychiatric Inpatient Hospitals. *J Psychiatr Pract* **2016**, *22*, 283-297.
35. Tesfaye, S.; Debencho, N.; Kisi, T.; Tareke, M. Prevalence of Antipsychotic Polypharmacy and Associated Factors among Outpatients with Schizophrenia Attending Amanuel Mental Specialized Hospital, Addis Ababa, Ethiopia. *Psychiatry J* **2016**, *2016*, 6191074.
36. Tiihonen, J.; Mittendorfer-Rutz, E.; Torniainen, M.; Alexanderson, K.; Tanskanen, A. Mortality and Cumulative Exposure to Antipsychotics, Antidepressants, and Benzodiazepines in Patients With Schizophrenia: An Observational Follow-Up Study. *Am J Psychiatry* **2016**, *173*, 600-606.
37. Gaviria, A.M.; Franco, J.G.; Aguado, V.; Rico, G.; Labad, J.; de Pablo, J.; Vilella, E. A Non-Interventional Naturalistic Study of the Prescription Patterns of Antipsychotics in Patients with Schizophrenia from the Spanish Province of Tarragona. *PLoS One* **2015**, *10*, e0139403.

38. Sneider, B.; Pristed, S.G.; Correll, C.U.; Nielsen, J. Frequency and correlates of antipsychotic polypharmacy among patients with schizophrenia in Denmark: A nation-wide pharmacoepidemiological study. *Eur Neuropsychopharmacol* **2015**, *25*, 1669-1676.
39. Nasrallah, H.A.; Harvey, P.D.; Casey, D.; Csoboth, C.T.; Hudson, J.I.; Julian, L.; Lentz, E.; Nuechterlein, K.H.; Perkins, D.O.; Kotowsky, N.; et al. The Management of Schizophrenia in Clinical Practice (MOSAIC) Registry: a focus on patients, caregivers, illness severity, functional status, disease burden and healthcare utilization. *Schizophr Res* **2015**, *166*, 69-79.
40. Li, Q.; Xiang, Y.T.; Su, Y.A.; Shu, L.; Yu, X.; Chiu, H.F.; Correll, C.U.; Ungvari, G.S.; Lai, K.Y.; Ma, C.; et al. Antipsychotic polypharmacy in schizophrenia patients in China and its association with treatment satisfaction and quality of life: findings of the third national survey on use of psychotropic medications in China. *Aust N Z J Psychiatry* **2015**, *49*, 129-136.
41. Fisher, M.D.; Reilly, K.; Isenberg, K.; Villa, K.F. Antipsychotic patterns of use in patients with schizophrenia: polypharmacy versus monotherapy. *BMC Psychiatry* **2014**, *14*, 341.
42. Xue, Q.; Xiong, X.; Feng, Y.; Yao, L.; Chen, S.; Xiang, L. Socioeconomic factors influencing antipsychotic prescription for schizophrenia inpatients in China: a cross-sectional study. *Int Clin Psychopharmacol* **2014**, *29*, 288-295.
43. Roh, D.; Chang, J.G.; Kim, C.H.; Cho, H.S.; An, S.K.; Jung, Y.C. Antipsychotic polypharmacy and high-dose prescription in schizophrenia: a 5-year comparison. *Aust N Z J Psychiatry* **2014**, *48*, 52-60.
44. Xiang, Y.T.; Ungvari, G.S.; Wang, C.Y.; Si, T.M.; Lee, E.H.; Chiu, H.F.; Lai, K.Y.; He, Y.L.; Yang, S.Y.; Chong, M.Y.; et al. Adjunctive antidepressant prescriptions for hospitalized patients with schizophrenia in Asia (2001-2009). *Asia Pac Psychiatry* **2013**, *5*, E81-87.
45. Banerjee, I.; Roy, B.; Sathian, B.; Banerjee, I.; Chakraborty, P.K.; Saha, A. Socio demographic profile and utilization pattern of antipsychotic drugs among schizophrenic inpatients: a cross sectional study from western region of Nepal. *BMC Psychiatry* **2013**, *13*, 96.
46. Goren, J.L.; Meterko, M.; Williams, S.; Young, G.J.; Baker, E.; Chou, C.H.; Kilbourne, A.M.; Bauer, M.S. Antipsychotic prescribing pathways, polypharmacy, and clozapine use in treatment of schizophrenia. *Psychiatr Serv* **2013**, *64*, 527-533.
47. Wu, C.S.; Lin, Y.J.; Feng, J. Trends in treatment of newly treated schizophrenia-spectrum disorder patients in Taiwan from 1999 to 2006. *Pharmacoepidemiol Drug Saf* **2012**, *21*, 989-996.
48. Himelhoch, S.; Slade, E.; Kreyenbuhl, J.; Medoff, D.; Brown, C.; Dixon, L. Antidepressant prescribing patterns among VA patients with schizophrenia. *Schizophr Res* **2012**, *136*, 32-35.
49. Xiang, Y.T.; Wang, C.Y.; Si, T.M.; Lee, E.H.; He, Y.L.; Ungvari, G.S.; Chiu, H.F.; Yang, S.Y.; Chong, M.Y.; Tan, C.H.; et al. Antipsychotic polypharmacy in inpatients with schizophrenia in Asia (2001-2009). *Pharmacopsychiatry* **2012**, *45*, 7-12.
50. Mundt, A.P.; Aichberger, M.C.; Fakhriddinov, S.; Fayzirahmanova, M.; Grohmann, R.; Heinz, A.; Ivens, S.; Magzumova, S.; Sartorius, N.; Strohle, A. Prescription patterns of patients diagnosed with schizophrenia in mental hospitals in Tashkent/Uzbekistan and in four German cities. *Pharmacoepidemiol Drug Saf* **2012**, *21*, 145-151.
51. Tsutsumi, C.; Uchida, H.; Suzuki, T.; Watanabe, K.; Takeuchi, H.; Nakajima, S.; Kimura, Y.; Tsutsumi, Y.; Ishii, K.; Imasaka, Y.; et al. The evolution of antipsychotic switch and polypharmacy in natural practice--a longitudinal perspective. *Schizophr Res* **2011**, *130*, 40-46.
52. Sim, K.; Yong, K.H.; Chan, Y.H.; Tor, P.C.; Xiang, Y.T.; Wang, C.Y.; Lee, E.H.; Fujii, S.; Yang, S.Y.; Chong, M.Y.; et al. Adjunctive mood stabilizer treatment for hospitalized schizophrenia patients: Asia psychotropic prescription study (2001-2008). *Int J Neuropsychopharmacol* **2011**, *14*, 1157-1164.
53. Nielsen, J.; le Quach, P.; Emborg, C.; Foldager, L.; Correll, C.U. 10-year trends in the treatment and outcomes of patients with first-episode schizophrenia. *Acta Psychiatr Scand* **2010**, *122*, 356-366.
54. Okumura, Y.; Ito, H.; Kobayashi, M.; Mayahara, K.; Matsumoto, Y.; Hirakawa, J. Prevalence of diabetes and antipsychotic prescription patterns in patients with schizophrenia: a nationwide retrospective cohort study. *Schizophr Res* **2010**, *119*, 145-152.
55. Kroken, R.A.; Johnsen, E.; Ruud, T.; Wentzel-Larsen, T.; Jorgensen, H.A. Treatment of schizophrenia with antipsychotics in Norwegian emergency wards, a cross-sectional national study. *BMC Psychiatry* **2009**, *9*, 24.

56. Olfson, M.; Marcus, S.C.; Wan, G.J. Treatment patterns for schizoaffective disorder and schizophrenia among Medicaid patients. *Psychiatr Serv* **2009**, *60*, 210-216.
57. Sim, K.; Su, H.C.; Fujii, S.; Yang, S.Y.; Chong, M.Y.; Ungvari, G.; Si, T.; He, Y.L.; Chung, E.K.; Chan, Y.H.; et al. High-dose antipsychotic use in schizophrenia: a comparison between the 2001 and 2004 Research on East Asia Psychotropic Prescription (REAP) studies. *Br J Clin Pharmacol* **2009**, *67*, 110-117.
58. Citrome, L.; Reist, C.; Palmer, L.; Montejano, L.; Lenhart, G.; Cuffel, B.; Harnett, J.; Sanders, K.N. Dose trends for second-generation antipsychotic treatment of schizophrenia and bipolar disorder. *Schizophr Res* **2009**, *108*, 238-244.
59. Xiang, Y.T.; Weng, Y.Z.; Leung, C.M.; Tang, W.K.; Ungvari, G.S. Long-term use of mood stabilizers and its impact on the quality of life of chinese patients with schizophrenia. *Clin Neuropharmacol* **2009**, *32*, 16-21.
60. dosReis, S.; Johnson, E.; Steinwachs, D.; Rohde, C.; Skinner, E.A.; Fahey, M.; Lehman, A.F. Antipsychotic treatment patterns and hospitalizations among adults with schizophrenia. *Schizophr Res* **2008**, *101*, 304-311.
61. Chen, H.; Kennedy, W.K.; Dorfman, J.H.; Fincham, J.E.; Reeves, J.; Martin, B.C. The effect of adjunctive mood stabilizers on antipsychotic utilization pattern and health resource utilization for Medicaid enrollees with schizophrenia. *Curr Med Res Opin* **2007**, *23*, 1351-1365.
62. Xiang, Y.T.; Weng, Y.Z.; Leung, C.M.; Tang, W.K.; Ungvari, G.S. Clinical and social determinants of antipsychotic polypharmacy for Chinese patients with schizophrenia. *Pharmacopsychiatry* **2007**, *40*, 47-52.
63. Megna, J.L.; Kunwar, A.R.; Mahlotra, K.; Sauro, M.D.; Devitt, P.J.; Rashid, A. A study of polypharmacy with second generation antipsychotics in patients with severe and persistent mental illness. *J Psychiatr Pract* **2007**, *13*, 129-137.
64. Kreyenbuhl, J.A.; Valenstein, M.; McCarthy, J.F.; Ganoczy, D.; Blow, F.C. Long-term antipsychotic polypharmacy in the VA health system: patient characteristics and treatment patterns. *Psychiatr Serv* **2007**, *58*, 489-495.
65. Shi, L.; Ascher-Svanum, H.; Zhu, B.; Faries, D.; Montgomery, W.; Marder, S.R. Characteristics and use patterns of patients taking first-generation depot antipsychotics or oral antipsychotics for schizophrenia. *Psychiatr Serv* **2007**, *58*, 482-488.
66. Mao, P.X.; Tang, Y.L.; Wang, Z.M.; Jiang, F.; Gillespie, C.F.; Cai, Z.J. Antipsychotic drug use in 503 Chinese inpatients with schizophrenia. *Int J Psychiatry Clin Pract* **2007**, *11*, 29-35.
67. Davids, E.; Bunk, C.; Specka, M.; Gastpar, M. Psychotropic drug prescription in a psychiatric university hospital in Germany. *Prog Neuropsychopharmacol Biol Psychiatry* **2006**, *30*, 1109-1116.
68. Hanssens, L.; De Hert, M.; Wampers, M.; Reginster, J.Y.; Peuskens, J. Pharmacological treatment of ambulatory schizophrenic patients in Belgium. *Clin Pract Epidemiol Ment Health* **2006**, *2*, 11.
69. Wheeler, A.; Humberstone, V.; Robinson, G. Trends in antipsychotic prescribing in schizophrenia in Auckland. *Australasian Psychiatry* **2006**, *14*, 169-174.
70. Kreyenbuhl, J.; Valenstein, M.; McCarthy, J.F.; Ganoczy, D.; Blow, F.C. Long-term combination antipsychotic treatment in VA patients with schizophrenia. *Schizophr Res* **2006**, *84*, 90-99.
71. Burns, T.; Christova, L.; Cooper, S.; Harrison, G.; McKendrick, J.; Laugharne, R.; Obuaya, T.; McCreadie, R.; O'Brien, S.; Perrington, S.; et al. Maintenance antipsychotic medication patterns in outpatient schizophrenia patients: a naturalistic cohort study. *Acta Psychiatr Scand* **2006**, *113*, 126-134.
72. Haro, J.M.; Novick, D.; Belger, M.; Jones, P.B. Antipsychotic type and correlates of antipsychotic treatment discontinuation in the outpatient treatment of schizophrenia. *Eur Psychiatry* **2006**, *21*, 41-47.
73. De Hert, M.; Wampers, M.; Peuskens, J. Pharmacological treatment of hospitalised schizophrenic patients in Belgium. *Int J Psychiatry Clin Pract* **2006**, *10*, 285-290.
74. Novick, D.; Bousono, M.; Suarez, D.; Olivares, J.M.; Montejo, A.L.; Haro, J.M.; Edgell, E.T.; Ratcliffe, M.; Board, S.A. Use of concomitant medication with antipsychotic treatment in outpatients with schizophrenia: results from the European Schizophrenia Outpatients Health Outcomes (SOHO) study. *Prog Neuropsychopharmacol Biol Psychiatry* **2005**, *29*, 972-982.
75. Edlinger, M.; Hausmann, A.; Kemmler, G.; Kurz, M.; Kurzthaler, I.; Walch, T.; Walpoth, M.; Fleischhacker, W.W. Trends in the pharmacological treatment of patients with schizophrenia over a 12 year observation period. *Schizophr Res* **2005**, *77*, 25-34.

76. Faries, D.; Ascher-Svanum, H.; Zhu, B.; Correll, C.; Kane, J. Antipsychotic monotherapy and polypharmacy in the naturalistic treatment of schizophrenia with atypical antipsychotics. *BMC Psychiatry* **2005**, *5*, 26.
77. Hodgson, R.; Belgamwar, R.; Al-tawarah, Y.; MacKenzie, G. The use of atypical antipsychotics in the treatment of schizophrenia in North Staffordshire. *Hum Psychopharmacol* **2005**, *20*, 141-147.
78. Magliano, L.; Fiorillo, A.; Guarneri, M.; Marasco, C.; De Rosa, C.; Malangone, C.; Maj, M.; National Mental Health Project Working, G. Prescription of psychotropic drugs to patients with schizophrenia: an Italian national survey. *Eur J Clin Pharmacol* **2004**, *60*, 513-522.
79. Ganguly, R.; Kotzan, J.A.; Miller, L.S.; Kennedy, K.; Martin, B.C. Prevalence, trends, and factors associated with antipsychotic polypharmacy among Medicaid-eligible schizophrenia patients, 1998-2000. *J Clin Psychiatry* **2004**, *65*, 1377-1388.
80. Sim, K.; Su, A.; Leong, J.Y.; Yip, K.; Chong, M.Y.; Fujii, S.; Yang, S.; Ungvari, G.S.; Si, T.; Chung, E.K.; et al. High dose antipsychotic use in schizophrenia: findings of the REAP (research on east Asia psychotropic prescriptions) study. *Pharmacopsychiatry* **2004**, *37*, 175-179.
81. Sim, K.; Su, A.; Fujii, S.; Yang, S.Y.; Chong, M.Y.; Ungvari, G.S.; Si, T.; Chung, E.K.; Tsang, H.Y.; Chan, Y.H.; et al. Antipsychotic polypharmacy in patients with schizophrenia: a multicentre comparative study in East Asia. *Br J Clin Pharmacol* **2004**, *58*, 178-183.
82. De las Cuevas, C.; Sanz, E.J. Polypharmacy in psychiatric practice in the Canary Islands. *BMC Psychiatry* **2004**, *4*, 18.
83. Rothbard, A.B.; Kuno, E.; Foley, K. Trends in the rate and type of antipsychotic medications prescribed to persons with schizophrenia. *Schizophr Bull* **2003**, *29*, 531-540.
84. Loosbrock, D.L.; Zhao, Z.; Johnstone, B.M.; Morris, L.S. Antipsychotic medication use patterns and associated costs of care for individuals with schizophrenia. *J Ment Health Policy Econ* **2003**, *6*, 67-75.
85. dosReis, S.; Zito, J.M.; Buchanan, R.W.; Lehman, A.F. Antipsychotic dosing and concurrent psychotropic treatments for Medicaid-insured individuals with schizophrenia. *Schizophr Bull* **2002**, *28*, 607-617.
86. Ungvari, G.S.; Chung, Y.G.; Chee, Y.K.; Fung-Shing, N.; Kwong, T.W.; Chiu, H.F. The pharmacological treatment of schizophrenia in Chinese patients: a comparison of prescription patterns between 1996 and 1999. *Br J Clin Pharmacol* **2002**, *54*, 437-444.
87. Clark, R.E.; Bartels, S.J.; Mellman, T.A.; Peacock, W.J. Recent trends in antipsychotic combination therapy of schizophrenia and schizoaffective disorder: implications for state mental health policy. *Schizophr Bull* **2002**, *28*, 75-84.
88. Meyer, P.S.; Bond, G.R.; Tunis, S.L.; McCoy, M.L. Comparison between the effects of atypical and traditional antipsychotics on work status for clients in a psychiatric rehabilitation program. *J Clin Psychiatry* **2002**, *63*, 108-116.
